# IgM-mediated protection drives early B-cell activation and mucosal containment of *Vibrio anguillarum* in Atlantic cod (*Gadus morhua)*

**DOI:** 10.3389/fimmu.2026.1771403

**Published:** 2026-03-06

**Authors:** Naomi Croft Guslund, Alexandra Jonsson, Anders K. Krabberød, Adrián López-Porras, Simen F. Nørstebø, Henning Sørum, Kjetill S. Jakobsen, Finn-Eirik Johansen, Shuo-Wang Qiao

**Affiliations:** 1 Section for Physiology and Cell Biology, Department of Biosciences, University of Oslo, Oslo, Norway; 2 Section for Genetics and Evolutionary Biology, Department of Biosciences, University of Oslo, Oslo, Norway; 3 Department of Preclinical Sciences and Pathology, Faculty of Veterinary Medicine, Norwegian University of Life Sciences, Ås, Norway; 4 Department of Paraclinical Sciences, Faculty of Veterinary Medicine, Norwegian University of Life Sciences, Ås, Norway; 5 Centre for Ecological and Evolutionary Synthesis, Department of Biosciences, University of Oslo, Oslo, Norway; 6 Department of Immunology, Institute of Clinical Medicine, University of Oslo, Oslo, Norway

**Keywords:** Atlantic cod, IgM, innate immunity, single-cell RNA sequencing, vaccination, *Vibrio anguillarum*, B cells

## Abstract

**Background:**

Atlantic cod lack functional MHC class II and CD4, raising fundamental questions about how vaccination generates protection in this species.

**Methods:**

We combined single-cell transcriptomic profiling of splenic cells with qRT-PCR across complementary active vaccination and passive serum-transfer experiments to define cellular and transcriptional correlates of immunity to *Vibrio anguillarum*.

**Results:**

Bath-vaccinated fish and recipients of immune serum showed effective containment of infection, with bacterial signals largely restricted to gills and minimal detection in spleen or head kidney, whereas naïve fish frequently developed high systemic bacterial loads by day 3 post-infection. All groups exhibited increased splenic macrophage abundance following challenge, but only naïve fish showed strong and sustained inflammatory activation, consistent with their higher pathogen burden. Vaccinated fish and immune-serum recipients displayed only transient or weak myeloid responses despite similar early neutrophil activation. Across both experimental models, a transcriptionally distinct B-cell subset expanded at the peak of infection. This population showed increased immunoglobulin and MHC class I expression together with innate sensing features, consistent with an activated B-cell state. Although this B-cell subset increased in all groups, the largest expansions were observed in vaccinated fish and immune-serum recipients. Overall, these findings are consistent with antigen-specific IgM enhancing early B-cell activation and contributing to protection against *V. anguillarum* through coordinated humoral and innate-like B-cell responses.

**Discussion:**

These findings identify an antibody-driven mode of immune coordination that operates independently of classical CD4^+^ T-cell help and provide insight into how effective vaccination can be achieved in vertebrates with divergent adaptive immune architectures.

## 1 Introduction

The Atlantic cod (*Gadus morhua*) possesses a highly unusual immune system that departs from classical vertebrate paradigms. In particular, cod lacks functional MHC class II and CD4 genes (1, 2), elements regarded as essential for helper T cell-mediated adaptive immunity. Despite this seemingly major immunological loss, cod as a species is not uniquely disease-prone under natural conditions, suggesting the evolution of alternative immune strategies (3). The absence of these canonical components has sparked interest in how cod mount protective immune responses, including responses induced by vaccination.

Previous studies in cod have reported weak or inconsistent specific antibody responses following immunization or infection, raising doubts about the role of IgM as a principal protective correlate (4–6). More recently, however, serum-transfer experiments using both crude serum and purified IgM from bath-immunized donors demonstrated that antigen-specific IgM was sufficient to confer protection against *Vibrio anguillarum*, providing strong evidence that IgM is indeed one of the major protective agents in this system (7). The present study analyses material from those same bath-vaccination and serum-transfer experiments published by Jonsson et al. (7), generating new insights.

Advances in single-cell RNA sequencing (scRNA-seq) have broadened the characterization of cod immune cell diversity. Through these approaches, splenic immune cell populations have been described to include lymphocytes, macrophages, granulocytes, dendritic cell (DC)-like cells, and GATA3^+^ cytotoxic cells proposed to function as innate lymphoid cell (ILC) equivalents (8, 9). Notably, more than 65% of splenic T cells have been reported to lack *cd8* expression based on transcriptomic profiling (9), indicating distinct adaptations within the cod lymphocyte repertoire. These observations emphasize the need to define how cellular immune responses in cod are coordinated, particularly in relation to vaccination and antigen-specific antibody production.

*V. anguillarum* is an opportunistic marine pathogen that causes vibriosis and ulcer disease in cod aquaculture, leading to systemic infection and substantial economic losses (10). To investigate the mechanisms underlying protection against *V. anguillarum* O2a (*Va*-O2a), two complementary infection timelines were analyzed: one following active bath vaccination with formalin-inactivated bacteria, and one following passive serum-transfer using immune serum from vaccinated donors. We used scRNA-seq on more than 100,000 splenic cells, combined with targeted quantitative reverse transcription PCR (qRT-PCR) assays for immune and bacterial genes in spleen, head kidney, and gill, and thereby captured both systemic and mucosal responses across vaccination and infection stages.

In this work, we describe the temporal dynamics of immune cell responses during infection and profile the transcriptional activity of immune cell subsets. Vaccinated fish exhibited strong protection, with *Va*-O2a largely contained at the mucosal surface and minimal dissemination to internal organs. Macrophage populations expanded in both naïve and vaccinated groups, but only naïve fish showed sustained inflammatory activation. A subset of activated B cells increased at the peak of infection in both experimental models, but more so in the vaccinated fish and the immune-serum recipients than in the naïve and naïve-serum recipients. No specific T-cell response or change in plasma-cell population size was detected. Together, these analyses identify immune correlates associated with protection against *V. anguillarum* and position Atlantic cod as a model in which antibody-dependent immune coordination can be examined in the absence of classical CD4^+^ T-cell help.

## 2 Methods

### 2.1 Overview of experimental material

**Figure 1:**
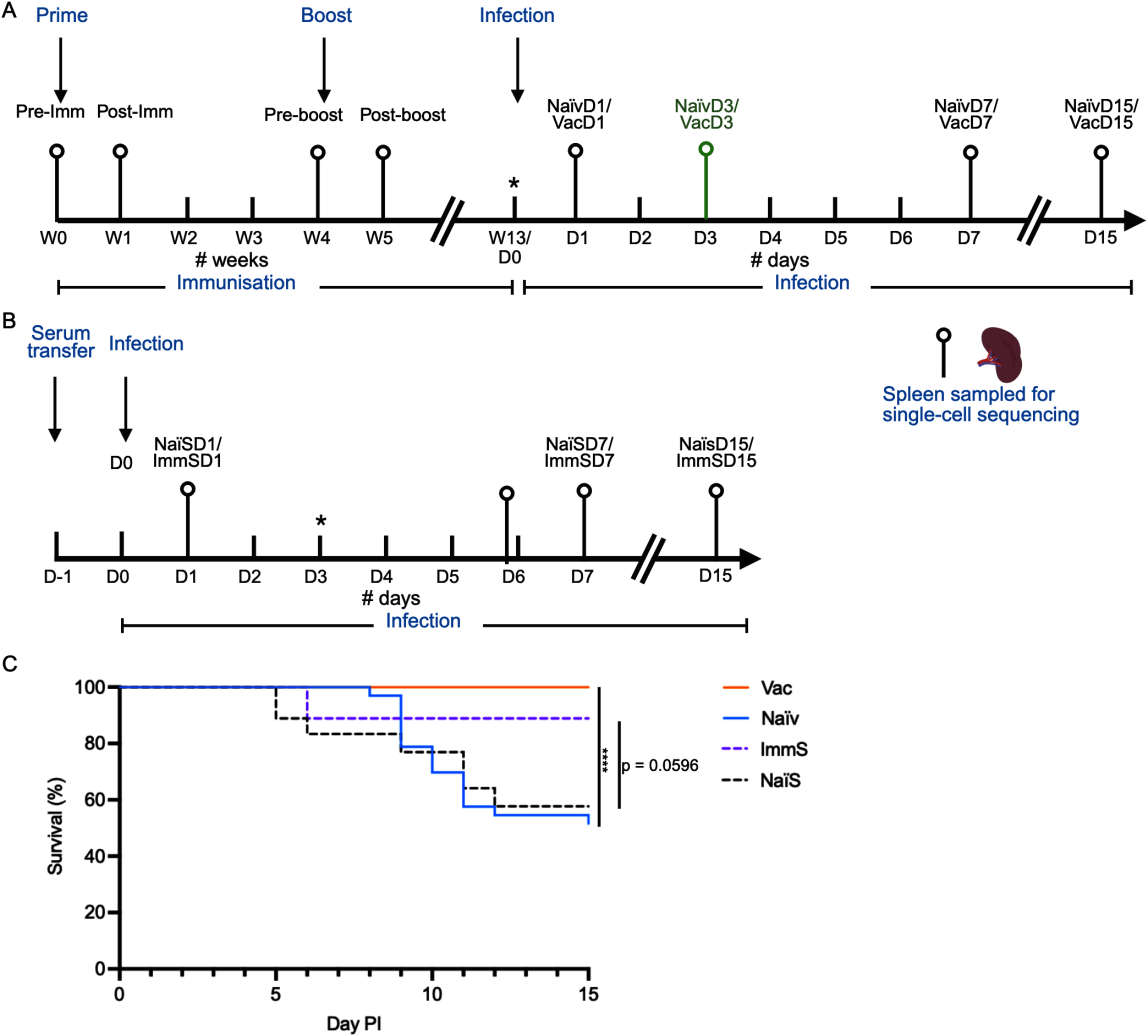
Overview of vaccination, immune challenge, sampling, and survival outcomes in Atlantic cod. **(A)** Active vaccination experiment. Each pinpoint represents a spleen sample taken from 2–3 fish for single-cell RNA sequencing (scRNA-seq). Fish were sampled prior to bath vaccination (Pre-Imm), 7 days after primary vaccination against *Vibrio anguillarum* O2a (*Va*-O2a) (Post-Imm), and three weeks later (Pre-boost). Fish then received a booster bath vaccination and were sampled 7 days later (Post-boost). Thirteen weeks after the first vaccination, both vaccinated (Vac) and naïve (Naïv) fish were exposed to Va-O2a by bath challenge on day 0 (D0) and were sampled on days (D) 1, 7, and 15 after infection. Samples collected three days after infection (VacD3 and NaïvD3) were obtained in a repeated experiment the following year. **(B)** Passive vaccination experiment. Immune serum was taken from vaccinated survivors of the active vaccination experiment, and naïve serum was collected from uninfected, naïve fish. Serum was injected on day −1, followed by infection on day 0. D0 fish were sampled prior to infection. Spleen samples for scRNA-seq were collected from immune serum (ImmS) and naïve serum (NaïS) recipients on days 1, 7, and 15 after infection. *Samples indicated by asterisks (D0 in panel***(A)****and D3 in panel****(B)****) were sequenced but yielded insufficient-quality data for downstream analysis.***(C)** Combined survival curves post infection (PI) from the two infectious-challenge experiments sampled for scRNA-seq. The continuous lines show survival of bath-vaccinated cod (Vac, n = 32, orange line) and naïve cod (Naïv, n = 33, blue line), adapted from **Figure 1** of Jonsson et al. (7), corresponding to the experiment described in **(A)**. The dotted lines show survival from a serum-transfer study adapted from **Supplementary Figure 3B**, Jonsson et al. (7), corresponding to the experiment described in **(B)**. In the study, naïve cod were injected with either naïve serum (NaïS, n = 16, black dotted line) or immune serum (ImmS, n = 16, purple dotted line) 24 hours before challenge. Statistical analysis of survival outcomes was conducted using the Mantel–Cox test, with significance indicated as follows: ****p < 0.0001.

The present study analyses tissue samples from two infection experiments previously reported in Jonsson et al. (7) (**Figure 1**): a bath vaccination–challenge experiment (Methods sections 2.2 and 2.4) and a crude serum-transfer experiment (Methods sections 2.3 and 2.4). Full operational details for the vaccination, serum collection and challenge procedures are available in Jonsson et al. All procedures were carried out at the NIVA Research Facility (Solbergstrand, Norway) under approval from the Norwegian Food Safety Authority and FOTS ID 21758.

### 2.2 Active vaccination by bath-challenge

Juvenile Atlantic cod were bath-vaccinated with formalin-inactivated *V. anguillarum* O2a (*Va*-O2a) using a prime and booster regimen and were PIT-tagged and maintained under standard seawater conditions.

### 2.3 Serum-transfer experiment

In the serum-transfer experiment, immune donor serum was collected from fish that had undergone *Va*-O2a bath vaccination and had survived the live-bacterial challenge, while naïve donor serum was collected from uninfected and unvaccinated cod. Naïve recipients, reared under identical conditions, were injected intraperitoneally with 0.3 ml of pooled crude immune serum (n = 22) or pooled naïve serum (n = 22) 24 hours before immersion challenge with live *Va*-O2a. Three fish per group were sampled for scRNA-seq on day 1 and a further three on day 7 post-infection; these individuals were excluded from the survival analysis.

### 2.4 Immersion challenge with *Vibrio anguillarum* O2a

All bacterial challenges were performed by immersion in seawater containing *Va*-O2a at a concentration of 1 × 10^7 CFU ml^−^¹ for the post-vaccination challenge, and at 5 × 10^7 CFU ml^−^¹ for the serum-transfer challenge. The challenge dosage for the serum transfer experiment was increased as earlier experiments revealed lower than expected mortality in naïve fish. Fish remained in the static, aerated suspension for one hour before normal tank flow was restored. Naïve (n = 39) and vaccinated (n = 38) fish were co-housed in the same tank and received identical infectious challenge in the bath vaccination experiment. Likewise, naïve-serum and immune-serum recipients were kept in the same tank during the exposure to live bacteria in the serum-transfer experiment. Six fish in each group were removed for scRNA-seq after infection, and thus not included in the survival count. Cod were challenged and maintained at a water temperature between 8 and 12 °C, corresponding to the ambient temperatures typically experienced at approximately 40 m depth in their natural habitat.

### 2.5 Sampling for scRNA-seq and qRT-PCR

An overview of sampling for scRNA-seq in the bath vaccination and serum-transfer experiments is shown in **Figures 1A, B**, respectively. Spleen samples for scRNA-seq were collected before and after primary vaccination (PreImm and PostImm), before and after booster vaccination (PreBoost and PostBoost), and at days 1, 7 and 15 after *Va*-O2a challenge (NaïvD1, NaïvD7, NaïvD15; VacD1, VacD7, VacD15). Preliminary scRNA-seq analysis indicated that early transcriptional responses were insufficiently captured, and the infection component was therefore repeated in a subsequent year to obtain additional spleen samples at day 3 post infection (NaïvD3 and VacD3), in an experiment mimicking the initial experiment. In the serum-transfer experiment, spleens for scRNA-seq were collected at day 0 (pre-challenge) and at days 1, 3, 7 and 15 after challenge.

Fish were euthanized by cranial concussion, and sampling commenced immediately. Spleens were placed immediately into ice-cold PBS containing 0.01% BSA solution, gently dissociated through a 100 µm cell strainer (Falcon) and diluted to 200 cells/µl for droplet-based scRNA-seq. All cells were kept in regular microcentrifuge tubes to minimize cell loss and kept on ice for transport and laboratory work. Tissues of fish from the bath vaccination experiment were snap frozen or stored in RNA-stabilizing buffer at −80 °C until extraction for qRT-PCR.

### 2.6 Single-cell sequencing

ScRNA-seq was performed according to published protocols (8, 11, 12), and is described in detail in our previous work (9). In short, scRNA-seq was carried out using a droplet generator (Nadia from Dolomite Bio), which encapsulates single cells with barcoded oligo(dT)-coated beads inside a droplet for first strand cDNA synthesis. Sequencing libraries were constructed as previously described (8) and sequenced at the Norwegian Sequencing center at Oslo University Hospital. The data is available at the ENA repository with Accession number PRJEB47815 and ERP132119. The Drop-seq Core Computational Protocol (13) was followed to demultiplex the raw sequencing reads and to map the reads to the Atlantic cod genome, gadMor3 (RefSeq accession GCF_902167405.1) using STAR alignment (14), compiled with GCC 8.3.0. Multi-mapped reads were removed by limiting the number of alignments to 1 using the ‘outSAMmultNmax’ parameter. The resulting reads were summarized into gene expression matrices containing gene counts for each cell.

### 2.7 Data integration, quality control, and dimension reduction

Raw single-cell count matrices from individual fish were processed and integrated in Seurat (version 5.3.0) following established workflows (9, 15–18). Quality control was performed within RStudio to remove low-quality cells and technical artifacts. Cells were retained if they contained more than 150 and fewer than 3,000 detected genes (nFeature_RNA), between 150 and 4,000 total UMI counts (nCount_RNA), and less than 5% mitochondrial transcripts (percent.mt). Cells outside these thresholds, including potential multiplets and empty droplets, were excluded.

Data were log-normalized, variable features identified, and gene expression values scaled prior to principal component analysis (PCA). Integration across fish was performed using the canonical correlation analysis (CCA)–based workflow in Seurat (reduction = “integrated.cca”), followed by unsupervised dimension reduction using UMAP. Several sequencing samples from different fish were found to be of insufficient quality for downstream analysis, due to a reduced number of captured genes and/or increased signs of ambient RNA contamination. From the bath vaccination and infection challenge, the removed low-quality samples include all week-13/day-0 samples, one vaccinated fish on day 1 post infection (leaving two high-quality samples that day), and one naïve fish on day 3 post infection (leaving two high-quality samples). All day-3 post-infection samples from the serum-transfer study were also of poor quality and removed (**Figure 1B**).

Clusters were defined using shared nearest neighbor (SNN) modularity optimization and annotated according to differentially expressed marker genes between clusters (8, 9). A low-quality cluster showing diffuse expression patterns across top markers and reduced gene feature counts was removed following integration (**Supplementary Figures 1A, B**). This cluster, previously annotated as “Spleen Stroma” (9), likely represents ambient RNA contamination. The resulting dataset, following filtering, comprised 105,162 splenic cells spanning vaccination, serum-transfer and infection timelines. Two minor B-cell subclusters (266 cells in total) expressing thrombocyte- and erythrocyte-associated markers were also excluded, yielding 18,627 high-confidence B cells for downstream analysis (**Supplementary Figure 2**).

The day-3 samples (NaïvD3 and VacD3) collected in a repeated infection timeline the following year overlapped the existing embeddings across all major cell populations in the integrated UMAP (**Supplementary Figure 3**), indicating that their transcriptional profiles were consistent with the original dataset and that batch effects were minimal after integration.

### 2.8 Gene annotation and naming

Gene symbol identifiers from the *Gadus morhua* reference genome (gadMor3, NCBI RefSeq accession GCF_902167405.1) were used where available. Genes lacking assigned gene names and represented only by LOC identifiers were named based on their protein coding name. Duplicate gene names were differentiated by sequential numbering (e.g. *gene*, *gene.1*, *gene.2*). The original gene symbol or LOC identifier and their updated names used in this work are listed in **Supplementary Table 1**.

### 2.9 Module score generation and gene selection

To examine coordinated transcriptional responses within defined immune cell populations, custom gene modules were generated for each major cell type. Differentially expressed genes were first identified within the relevant cell cluster using Seurat’s FindMarkers function. From these, genes showing consistent and biologically interpretable expression changes across infection timepoints were manually selected to construct functional modules representing coherent biological processes. Gene selection was guided by published literature, allowing inclusion of duplicated genes and teleost-specific loci not captured by standard pathway resources.

Prior to module scoring, gene expression values were log-normalized and scaled within Seurat. Per-cell module scores were then calculated using Seurat’s AddModuleScore function, which computes the average scaled expression of genes within a module for each cell, relative to a control gene set. These per-cell module scores were subsequently summarized at the level of individual fish by averaging across all cells of the relevant type, yielding a single module score per fish for downstream comparison.

For visualization of gene-level patterns in heatmaps, expression values for individual genes were handled separately from module scores. For each gene, average expression values were calculated per fish within the relevant cell population and z-scored across fish to highlight relative up- or down-regulation over the experimental timeline. These gene-level z-scores were used exclusively for heatmap visualization and were not used in the calculation of module scores. Module names reflect the dominant biological processes represented by the selected genes and were applied consistently across active vaccination and passive immunization datasets.

### 2.10 RNA extraction, cDNA synthesis, and quantitative PCR

Total RNA was extracted from spleen, head kidney, and gill tissue using the RNeasy Plus Mini Kit (Qiagen, Germany) according to the manufacturer’s protocol. Tissue (<30 mg) was homogenized in RLT buffer containing 1% β-mercaptoethanol using a sterile syringe and needle, and genomic DNA was removed using gDNA Eliminator spin columns. RNA yield and purity were assessed by spectrophotometry (NanoDrop; Thermo Fisher Scientific), and a subset of samples was evaluated for integrity using a Bioanalyzer (Agilent Technologies), confirming RIN values > 6.

For host immune gene expression, cDNA was synthesized from ~500 ng total RNA using the SuperScript IV First-Strand Synthesis System (Thermo Fisher Scientific) with oligo(dT) primers. For bacterial gene detection (*toxR*, 16S rRNA), parallel reactions were prepared using random hexamer primers. The resulting cDNA was diluted before qRT-PCR as required to achieve comparable Cq values across samples.

Quantitative PCR was performed on a LightCycler 96 instrument (Roche) using SYBR Green I Master Mix (Roche) in 20 µL reactions. Primer specificity was confirmed by single-peak melt curves and the absence of amplification in no-template controls. All reactions used 45 amplification cycles and gene-specific annealing temperatures (59–63 °C), see **Supplementary Table 2** for primer details. All qRT-PCR reactions were performed in technical triplicate. Mean Cq values were calculated per sample after exclusion of obvious outlier replicates and exported for downstream analysis.

Relative expression values were calculated using an efficiency-corrected ΔΔCq approach, with *eef1a1* serving as a common reference gene to control variation in cDNA input across all assays. For each assay, mean Cq values were calculated per sample for the target gene and the reference gene. ΔCq was defined as Cq_target minus Cq_ref. A single calibrator sample (naïve fish, day 0) was used for all genes, and ΔΔCq values were obtained by subtracting the calibrator ΔCq from each sample’s ΔCq. Primer-specific amplification efficiencies (E) were taken from standard curve reactions (E = 1 + efficiency%/100) and applied directly in the expression calculation. Relative transcript abundance was computed as E^(−ΔΔCq), giving efficiency-corrected fold changes normalized to the calibrator. All plotted values correspond to these efficiency-adjusted relative expression estimates.

## 3 Results

### 3.1 Survival analysis

Survival patterns in the active vaccination bath-challenge and in serum-transfer groups are shown in **Figure 1C**. These are adapted from results shown in Jonsson et al. (7), and correspond to the fish from which scRNA-seq samples were obtained. Bath-vaccinated cod displayed complete protection following *Va*-O2a challenge, whereas naïve fish showed ~50% survival, a highly significant difference (log-rank test p < 0.0001). In the serum-transfer experiment immune-serum recipients showed improved, but not statistically significant survival relative to naïve-serum controls (log-rank p ≈ 0.059), probably due to the low numbers of fish included in this experiment due to a limited amount of immune serum. In a repeated, larger serum-transfer experiment (**Supplementary Figure 4**; (7), immune-serum recipients showed significantly improved survival of ~67% after *Va*-O2a challenge compared with naïve-serum recipients with only ~10% survival (log-rank test *p <* 0.0001), similar to that of naïve cod receiving no serum. Bath-vaccinated fish in this same infection experiment showed the highest survival rate at 90%.

### 3.2 Pathogen burden

**Figure 2:**
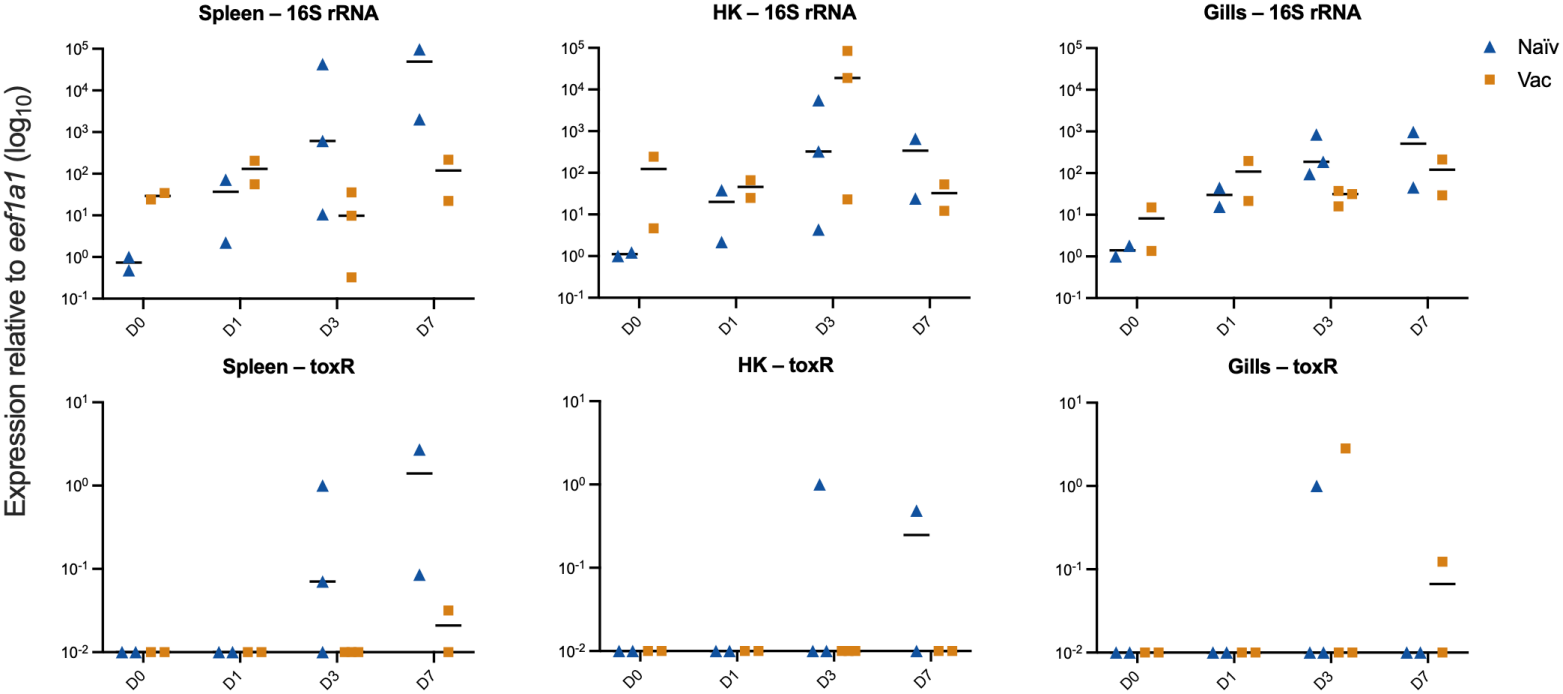
Bacterial 16S rRNA and *Vibrio anguillarum* toxR mRNA expression in spleen, head kidney (HK), and gills, as measured by qPCR. Expression is shown relative to host *eef1a1* expression (log_10_ scale) at days (D) 0, 1, 3, and 7 post-infection in naïve (blue triangles) and vaccinated (orange squares) fish. Each point represents one fish, and bars indicate medians. Data are from the bath-vaccination and infection experiment.

Pathogen burden was quantified by qRT-PCR targeting the 16S rRNA gene to estimate total bacterial presence, and *toxR* to specifically measure *V. anguillarum* presence (**Figure 2**). Clear between-group differences appeared in systemic organs from day 3. In spleen, two naïve fish showed very high 16S rRNA gene levels on day 3 (~600-fold and ~43,000-fold increase), while the third naïve fish remained low (~10-fold increase). It was this naïve fish (which expressed low levels of the 16S rRNA gene) that was removed from the scRNA-seq data due to low quality (methods section 2.8). In spleens from vaccinated cod, the 16S rRNA gene levels remained considerably lower, all less than 220-fold increase. Bacterial burden in head kidney samples showed the same split among the day 3 naïve fish: the two high-burden individuals reached ~330-fold and ~5,500-fold increase, whereas the low-burden naïve fish remained near baseline (~4-fold increase). Head kidney 16S rRNA gene levels from vaccinated fish were low at day 1 and day 7 (12–67-fold increase), but at day 3 two out of three examined fish showed substantial increases (~19,000-fold and ~85,000-fold) while the third remained low (~23-fold increase). In gills, both groups showed increased 16S rRNA gene levels after exposure, with overlapping values at day 1 and day 7. At day 3, naïve 16S rRNA gene gill values ranged from ~95-fold to ~840-fold increase, whereas vaccinated fish remained lower (~16–38-fold). The *toxR* assay aligned with the spleen and head kidney 16S rRNA gene expression patterns. In spleen and head kidney, *toxR* was detected in the naïve fish with the highest 16S rRNA gene levels on day 3 and day 7 after infection. Organs from vaccinated fish were *toxR*-negative except for one spleen with a low signal at day 7 after infection. In gills, *toxR* was detected in single individuals at day 3 in both groups and again at day 7 in vaccinated fish.

### 3.3 Splenic single-cell transcriptomic profiling

**Figure 3:**
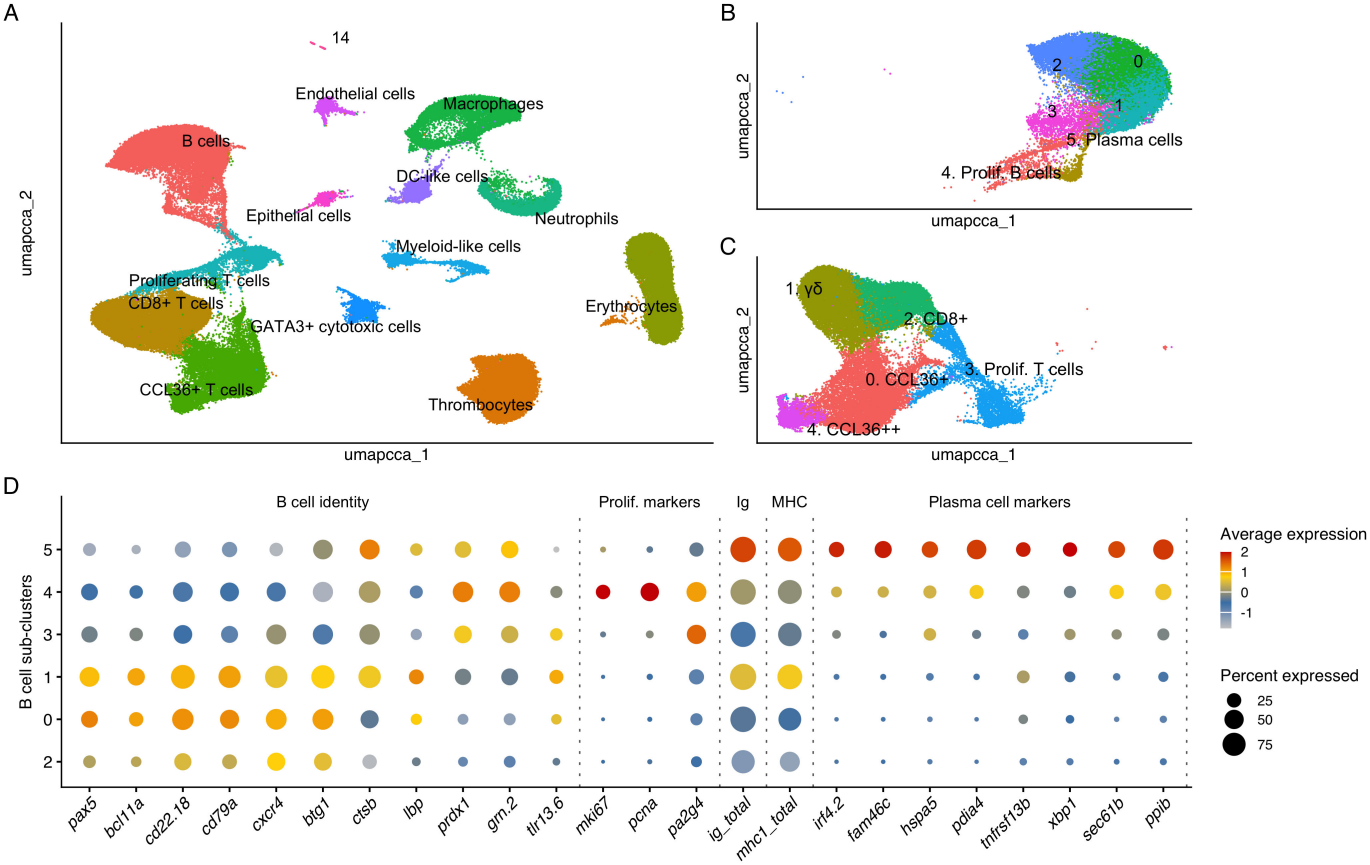
Global splenic cell landscape and B cell substructure in Atlantic cod. **(A)** UMAP of 105,162 splenic cells from the active and passive vaccination experiments. Each point represents a single cell positioned by transcriptomic similarity. Cluster identities were assigned based on differentially expressed marker genes. **(B, C)** Sub-clustering of lymphocyte populations showing 18,627 B cells and 30,505 T cells, respectively, and their associated sub-populations. Clusters 0–3 in the B-cell compartment remain unnamed to avoid over-interpretation. **(D)** DotPlot showing expression of selected markers across the six B-cell sub-clusters identified in panel **(B)** Dot size indicates the percentage of cells within each sub-cluster with detectable expression of the indicated feature. Dot color represents the mean log-normalized expression level within each sub-cluster, scaled per gene for visualization and therefore comparable across clusters but not between different genes. Genes are grouped by biological function and separated by dotted lines: a B-cell identity and homeostasis block; proliferation markers; summary features for Ig (ig_total, average per-cell expression of IGH and IGL genes detected in at least 5 percent of B cells) and MHC (mhc1_total, average per-cell expression of MHCI genes detected in at least 5 percent of B cells); and a plasma-cell differentiation and secretory program. Sub-clusters are ordered based on the pseudotime trajectory shown in **Supplementary Figure 5**.

Single-cell transcriptomic profiling of 105,162 splenic cells from the active and passive vaccination experiments revealed the expected major immune and non-immune cell types, consistent with earlier Atlantic cod datasets [**Figure 3A**; (8, 9)]. Fourteen clusters were identified based on differential expression of established marker genes, including B cells and T-cell populations, macrophages, neutrophils, myeloid-like cells, DC-like cells, thrombocytes, erythrocytes, endothelial cells, and epithelial cells. Cluster 14 represents ~0.1% of analyzed cells and remains unnamed.

Sub-clustering of lymphocytes separated 18,627 B cells and 30,505 T cells into transcriptionally distinct sub-clusters (**Figures 3B, C**). These sub-clusters are mostly consistent with the clustering reported by Guslund et al. (9), which described lymphocyte populations based on the same bath vaccination and infection challenge scRNA-seq data. However, that analysis did not include the serum-transfer scRNA-seq data that has been added here, which has more than doubled the number of lymphocytes analyzed. The previously described *cd8*-negative, MCP1b^+^ and MCP1b^+++^ populations are now renamed in this work as CCL36^+^ T cells and CCL36^++^ T cells to reflect the updated gadMor3 gene annotation of *mcp1b* (LOC115529242) to *ccl36.1* (19*)*. B-cell sub-clustering yielded six transcriptionally distinct groups (**Figure 3D**). Sub-cluster 5 showed a clear plasma-cell profile, differentially expressing multiple genes supporting this cluster’s identity (*irf4.2, fam46c, hspa5, pdia4, tnfrsf13b, xbp1, sec61b, ppib*), while sub-cluster 4 displayed a proliferation signature (*mki67, pcna, pa2g4)*. Both clusters match those described previously (9). Slight differences in the other B-cell clusters are shown compared to the prior published work, due to an increase in number of cells analyzed and improved filtering of ambient RNA.

B cell sub-cluster 1 was distinguished by the highest *ig_total* and *mhc1_total* expression after the plasma cells, together with a higher expression of innate sensing and processing genes such as *lbp*, *tlr13.6* and *ctsb* than sub-clusters 2, 0, 3 and 4. These changes occurred while core B-cell identity markers (*cd79a*, *cd79b*, *pax5*, *bcl11a*) remained highly expressed, consistent with an early activated or antigen-primed B-cell state rather than terminal differentiation. Sub-clusters 0 and 2 strongly expressed canonical B-cell identity genes and showed low expression of proliferation markers, consistent with the profile of a resting B-cell population. Sub-cluster 3 expressed these identity markers at lower levels and lacked the proliferative, secretory or activation-associated features seen in sub-clusters 4, 5 and 1 respectively.

### 3.4 Macrophage population change

**Figure 4:**
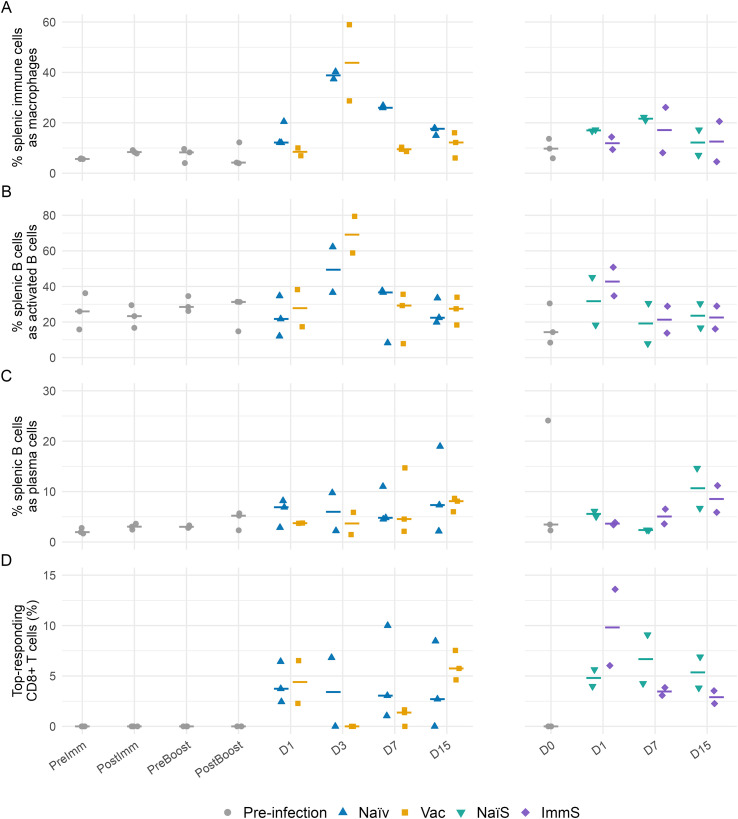
Relative proportions of immune cell subsets in Atlantic cod spleen across active vaccination and serum-transfer challenge timelines. Panels show the same timeline layout: active vaccination on the left and serum-transfer on the right. Fish were sampled before challenge (baseline) and at days (D) 1, 3, 7, and 15 post-challenge in the active vaccination experiment, and at day 0 and days 1, 7, and 15 post-challenge in the serum-transfer experiment. Each symbol represents an individual fish, colored and shaped by experimental group (baseline: gray circle; naïve: blue triangle; vaccinated: yellow square; naïve-serum recipients: teal triangle; immune-serum recipients: purple diamond). Horizontal bars indicate group medians. **(A)** Percentage of macrophages among all splenic immune cells. **(B)** Percentage of activated B cells (sub-cluster 1) among all splenic B cells. **(C)** Percentage of plasma cells (sub-cluster 5) among all splenic B cells. **(D)** Percentage of CD8+ T cells classified as top responders based on a cytotoxic/IFN-γ module, defined per side and day as exceeding the 95th percentile of the corresponding naïve reference (Naïv for the left timeline, NaïS for the right).

Measuring the population sizes of splenic immune cells revealed clear changes following infection marked by an early and sustained increase in the proportion of macrophages in naïve fish (**Figure 4A**). Percentages were calculated as the proportion of macrophages relative to the total splenic immune cell compartment, excluding erythrocytes, thrombocytes, endothelial and epithelial cells. Prior to infection (PreImm, PostImm, PreBoost, PostBoost and D0), macrophages comprised approximately 4–14% of splenic immune cells. In the bath-vaccination and challenge experiment (left panel of **Figure 4A**), one naïve fish showed an early rise to 20% macrophages at day 1, while the other naïve day 1 samples remained near baseline. The percentage of macrophages in all naïve fish increased to 37–40% on day 3, remained elevated at 26–27% on day 7 and declined to 15–18% on day 15. Vaccinated fish also increased at day 3 (29–59%) but returned close to baseline by day 7 (9–10%). In the serum-transfer experiment (right panel of **Figure 4A**), macrophage levels in naïve-serum recipients reached 17% on day 1, increased to 21–22% on day 7, and decreased to 7–17% on day 15. Immune-serum recipients showed 9–14% macrophages on day 1, similar to naïve-serum recipients at this timepoint. Variability within the immune-serum group increased at later timepoints, spanning 8–26% at day 7 and 5–21% at day 15, in contrast to the narrower ranges observed in naïve-serum recipients.

### 3.5 Lymphocyte response

Pseudotime analysis of B cells showed trajectory similar to previously published results (9), where the relatively large sub-cluster 2 representing naïve B cells became gradually more differentiated along the trajectory ending in either actively dividing cells in sub-cluster 4 or plasma cells in sub-cluster 5 (**Supplementary Figure 5**). We have focused on sub-cluster 1 that represented highly activated B cells, and of which the proportion showed large variation throughout the course of infection (**Figure 4B**). Prior to infection (PreImm, PostImm, PreBoost, PostBoost and day 0), sub-cluster 1 B cells represented 8–36% of the splenic B-cell compartment. In the bath-vaccination and challenge experiment (left panel of **Figure 4B**), sub-cluster 1 B cells showed a clear rise at day 3 post infection in both naïve and vaccinated fish: the sub-cluster 1 B cells in naïve fish reached 62% and 37% of all splenic B cells, and 79% and 59% in vaccinated fish. In the serum-transfer experiment (right panel of **Figure 4B**), a modest increase of sub-cluster 1 B cells was evident at day 1, with naïve-serum recipients at 45% and 18% of all splenic B cells, and immune-serum recipients at 51% and 35%.

The percentage of B cells classified as plasma cells (sub-cluster 5) remained low and did not show a consistent trend over time in either experiment (**Figure 4C**). Prior to infection (PreImm, PostImm, PreBoost, PostBoost and day 0), plasma cells generally comprised ~2–6% of splenic B cells, with one day 0 fish in the serum-transfer study reaching 24%. After challenge in the bath vaccination experiment, both naïve and vaccinated fish mostly remained within a similar range at all timepoints (~2–11%), with occasional higher values in individual fish (<19%) but without a systematic difference between naïve and vaccinated groups or across timepoints. In the serum-transfer experiment, naïve-serum and immune-serum recipients likewise showed plasma-cell frequencies largely within the same low range (~2–11%) at day 1, day 7 and day 15, again with no consistent divergence between groups. The percentage composition of the other B-cell and T-cell sub-clusters did not noticeably change over the infection timelines (**Supplementary Figure 6**, **7**).

To identify CD8^+^ T cells that were strongly responding to infection, we defined “top responders” as cells with very high expression of cytotoxic and IFN-γ–related genes (**Figure 4D**). For each timepoint after infection, we used the naïve response of non-vaccinated fish to define what a high response looked like by calculating the 95th percentile of the cytotoxic module score across all CD8^+^ T cells from non-vaccinated fish on that day. This value was then used as a fixed threshold. We subsequently calculated, for each individual fish, the percentage of its CD8 ^+^ T cells that exceeded this naïve-defined threshold. Because the cutoff was derived from all naïve cells pooled together, individual naïve fish are not expected to show exactly 5%. This analysis was designed to test whether prior antigen exposure through vaccination resulted in an increased frequency of highly cytotoxic CD8^+^ T cells after challenge. All pre-infection samples (PreImm, PostImm, PreBoost, PostBoost, and day 0) showed 0%, because the method relies on a same-day response to infection in non-vaccinated fish as reference to define the activation threshold, which does not exist before infection.

Across both experiments, the percentage of CD8^+^ T cells classified as top responders remained low. Following challenge in the bath vaccination experiment, naïve and vaccinated fish showed similar values across all days, largely ≤10%. In the serum-transfer experiment, both naïve-serum and immune-serum recipients also remained within this low range, apart from an increase in a single day 1 immune-serum recipient fish (<14%). Overall, no consistent group-specific differences were detected.

qRT-PCR assays were used to examine broader T- and B-cell activity across the infection timeline (**Supplementary Figures 8**, **9**). Transcript levels of *cd3g* (pan-T-cell marker) and the CCL36^++^ T cell–associated genes *ccl36* and *il18rap* showed no clear differences between naïve and vaccinated fish at any timepoint. Similarly, B cell-related genes (*cd22*, *cd79a*, and secreted *igm*) remained relatively stable across the timelines, with naïve and vaccinated groups displaying overlapping expression levels. Overall, these qRT-PCR measurements did not reveal group-specific shifts in total T-cell or CCL36^++^ T-cell abundance, or bulk B-cell responses.

### 3.6 Macrophage, DC-like and neutrophil responses

Because GSEA, KEGG pathways and similar resources are not adapted for non-model organisms such as the Atlantic cod and do not capture the duplicated pattern-recognition receptors (PRR) and MHCI families or several teleost-specific immune effectors, we developed small, curated pathway modules for each cell type to serve the same functional purpose in our analyses. These modules summarize coherent biological programs that are well-supported by the known relationships among the selected genes: a canonical inflammatory program in macrophages, PRR, toll-like receptor (TLR)-downstream and MHCI antigen-processing programs in DC-like cells, and antimicrobial or granule-associated effector programs in neutrophils. Module scoring allowed us to quantify coordinated transcriptional responses at the level of individual fish and to relate these functional changes to the cell-abundance patterns shown earlier.

**Figure 5:**
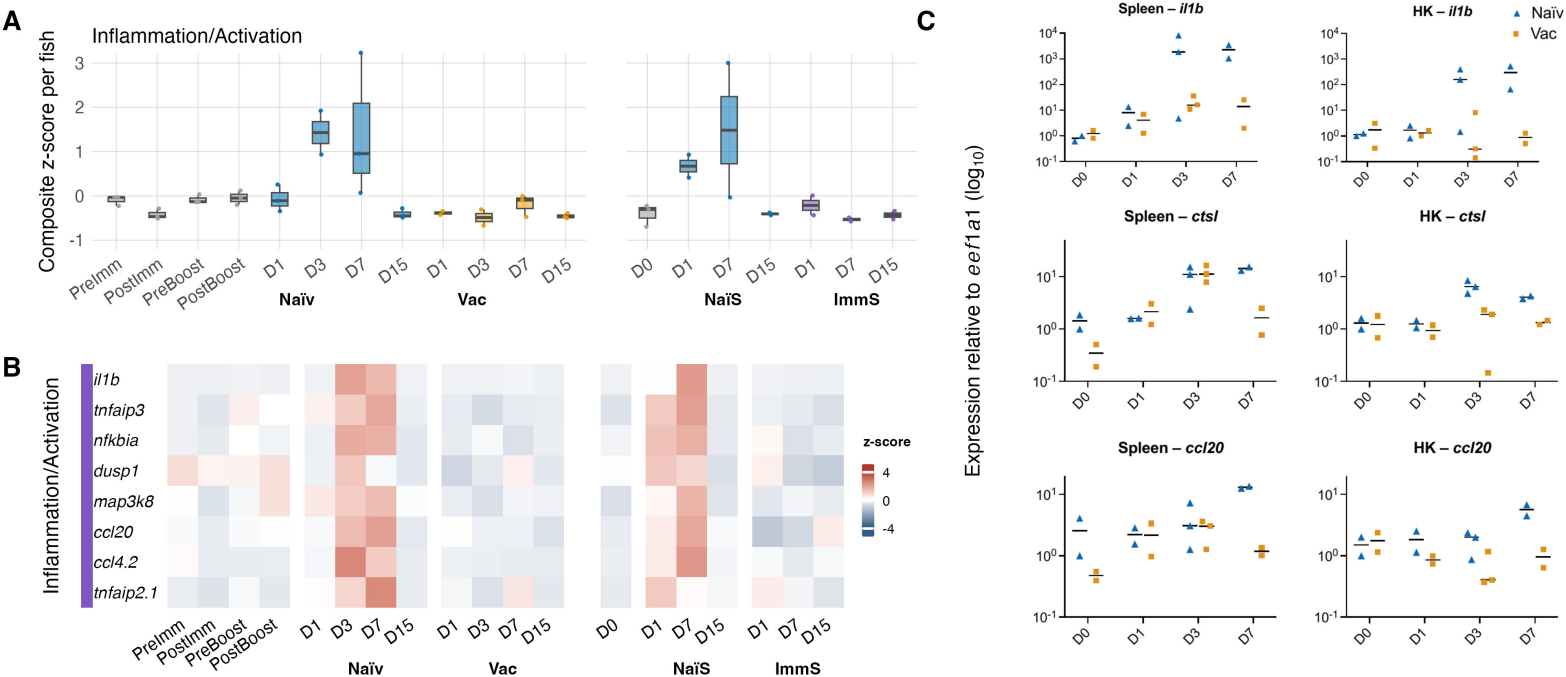
Macrophage inflammation modules and gene expression across infection timelines. **(A)** Inflammation/activation module scores in splenic macrophages across active and passive immunization experiments. The module (*il1b, tnfaip3, nfkbia, dusp1, map3k8, ccl20, ccl4.2, tnfaip2.1*) was calculated per fish as a composite z-score (mean expression per gene per fish, z-scored within gene across all groups, then averaged across genes). The left panel shows the active vaccination timeline (PreImm, PostImm, PreBoost, PostBoost) and post-challenge days (D) 1, 3, 7, and 15 for naïve (blue) and vaccinated (orange) fish. The right panel shows the passive serum-transfer experiment, with day 0 before challenge (gray) followed by post-challenge days 1, 7, and 15 for recipients of naïve serum (blue) or immune serum (purple). Points represent individual fish; boxplots summarize fish-level distributions. **(B)** Heatmap of the same inflammation/activation genes in splenic macrophages, showing relative expression patterns across the corresponding active and passive immunization timelines. Log-normalized RNA expression was averaged per gene and fish, z-scored within gene across groups, and averaged across fish to yield group-level mean z-scores (color scale: −4 to +4). Rows indicate individual genes belonging to the inflammation/activation module; columns represent experimental groups. Color intensity reflects relative expression within gene (not between genes). **(C)** Expression of macrophage-associated genes (*il1b, ctsl.4, ccl20*) in spleen and head kidney (HK) relative to *eef1a1* (log_10_) at days 0, 1, 3, and 7 post-infection in naïve (blue triangle) and vaccinated (orange square) fish from the active vaccination experiment. Each point represents a single fish; horizontal bars show group medians.

In the bath vaccination experiment, the splenic macrophages of naïve fish showed clear induction of the inflammation module after infection (**Figure 5A**), with module scores ranging from ~0.93–1.9 at day 3 and ~0.07–3.2 at day 7, matched by coordinated upregulation of all module genes in the heatmap (**Figure 5B**). These responses were notably variable, particularly at day 7 where individual naïve fish ranged from near-baseline to strongly elevated module activation. Vaccinated fish, in contrast, remained near or below zero at all post-challenge timepoints (~−0.67 to 0.00). A similar pattern occurred in the serum-transfer experiment: naïve-serum recipients showed elevated module scores at day 1 (~0.41–0.93) and day 7 (~−0.03–3.0), accompanied by corresponding increases in module-gene expression, whereas immune-serum recipients displayed little or no induction and stayed near or below baseline throughout (~−0.58 to 0.01).

In the scRNA-seq dataset, which contains only splenic cells, expression of *il1b*, *ctsl.4*, and *ccl20* was largely restricted to the macrophages. **Figure 5C** shows qRT-PCR expression of these genes in spleen and head kidney, with **Supplementary Figure 10** showing qRT-PCR data for the same genes in gills. In the systemic organs, *il1b* showed a strongly heterogeneous day-3 response in naïve fish: the same two high pathogen-burden individuals reached several-thousand-fold *il1b* increase in spleen and several-hundred-fold in head kidney, whereas the third naïve fish showed only modest induction (less than 5-fold in both organs). *Ctsl.4* also rose at day 3 in naïve fish, reaching ~10–15-fold in spleen in the individuals with high pathogen burden (and only 2.4-fold in the other), while in head kidney all three naïve fish showed a similar induction (~5–8-fold, including ~6.4-fold in the low-burden fish). Vaccinated fish showed low to moderate increases of *il1b* and *ctsl.4* at day 3 (<36-fold *il1b* and <17-fold *ctsl.4* in spleen; <8-fold *il1b* and <2.3-fold *ctsl.4* in head kidney). In the gills, responses were more uniform across individuals: naïve fish reached ~100–400-fold for *il1b* and ~7-fold for *ctsl.4* at day 3, whereas vaccinated fish remained low (<14-fold *il1b* and <5-fold *ctsl.4*). *Ccl20* remained low on day 3 and then rose in naïve fish at day 7 in spleen (~13-fold) and head kidney (~4–7). Gills showed a split response. In naïve fish, one individual showed high *ccl20* induction at day 3 and day 7 (~17-fold and ~12-fold), while the others showed only low to moderate increases (~1.6–4.6-fold). Vaccinated fish showed a similar pattern at day 7, with one fish reaching ~12-fold while the other remained near baseline (~1-fold). In conclusion, the macrophage expression of inflammation marker genes *il1b*, *ctsl.4*, and partially *ccl20*, seems to correlate closely with the bacterial load in the same organs. The expression level increased much more in naïve fish compared to vaccinated fish.

**Figure 6:**
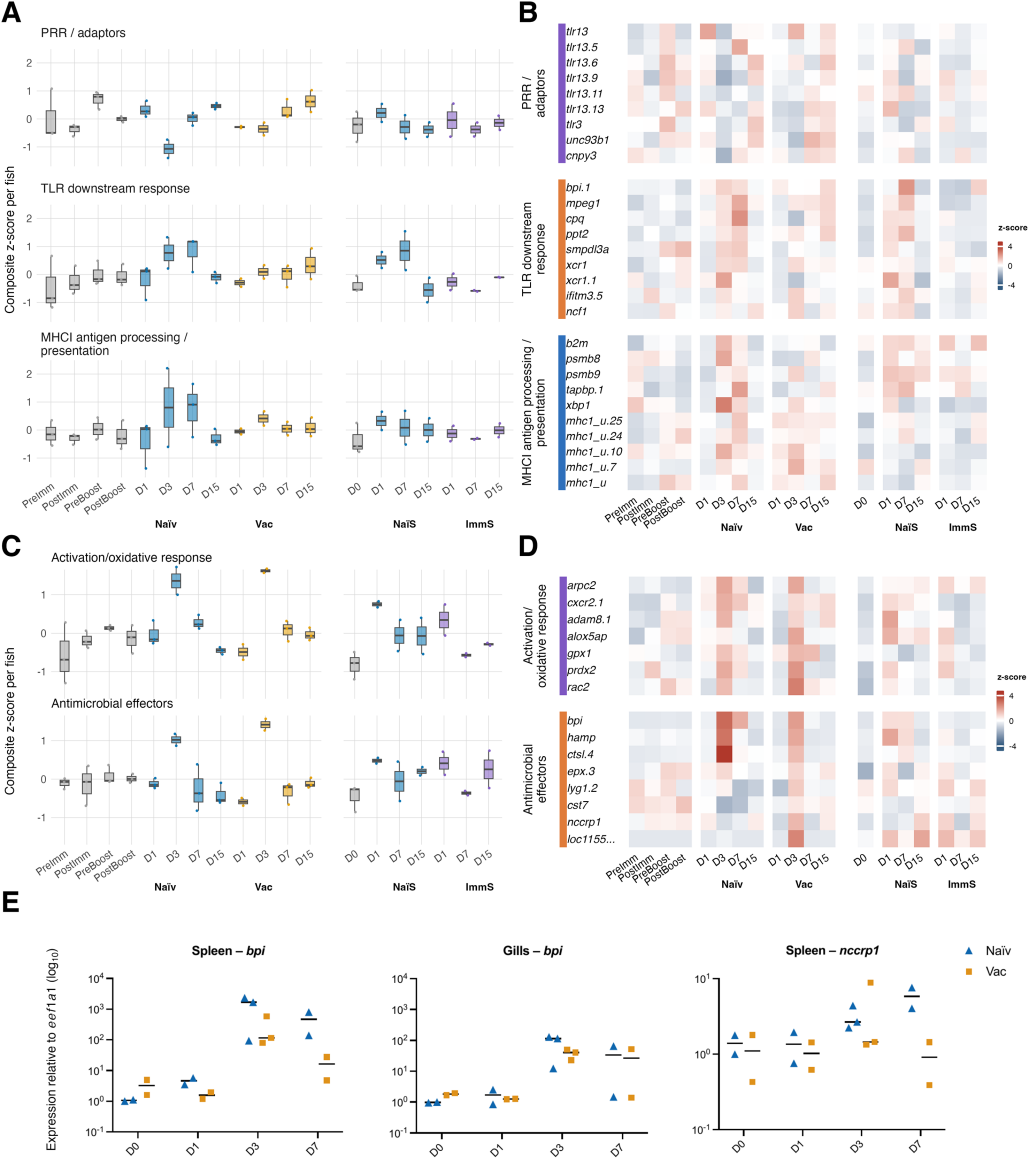
Module scores and gene expression in DC-like cells and neutrophils across infection timelines. Panels show results from the active vaccination experiment (PreImm, PostImm, PreBoost, PostBoost, followed by post challenge days (D) 1, 3, 7 and 15 in naïve and vaccinated fish) and the passive serum transfer experiment (day 0 control, followed by post challenge days 1, 7 and 15 in recipients of naïve serum [NaïS] or immune serum [ImmS]). Module scores represent composite z-scores calculated per fish (mean expression per gene per fish, z-scored within gene across all groups, then averaged across genes). Points represent individual fish and boxplots summarize fish level distributions. Heatmaps show gene level z-scored expression averaged per gene and fish. **(A)** Module scores for three functional modules in DC-like cells. These modules are directly derived from the genes shown in panel B. **(B)** Heatmap showing expression of the genes comprising the DC-like cell modules in panel A, grouped by module. **(C)** Module scores for two functional modules in neutrophils, calculated from the gene sets shown in panel D. **(D)** Heatmap of the neutrophil module genes corresponding to the modules in panel **(C)** The gene label “*loc1155…*” denotes *LOC115550932*, an aerolysin-like protein in the *Gadus morhua* genome assembly (gadMor3) that lacks an assigned gene symbol. **(E)** Expression of neutrophil associated genes in spleen and gills relative to *eef1a1* (log_10_). From left to right the panels show *bpi* expression in spleen, *bpi* expression in gills and *nccrp1* expression in spleen. Each point represents a single fish and horizontal bars show group medians. The timeline is from the active vaccination experiment.

PRR/adaptor, TLR-downstream response, and MHCI antigen-processing module scores in DC-like cells are shown in **Figures 6A**, while the corresponding gene-level z-scores are shown in **Figure 6B**. The PRR/adaptor module showed its strongest decrease at day 3 in naïve fish (module score −1.07), reflected by coordinated negative z-scores across nearly all *tlr13* paralogues, *tlr3*, *unc93b1*, and *cnpy3*. Several of these genes also showed reduced expression in vaccinated fish at day 3, but this did not translate into a clear change in the module score. The TLR-downstream response module was strongly induced at day 3 and day 7 in naïve fish (module scores +0.77 and +0.81). Positive z-scores were evident for *bpi.1*, *mpeg1*, *cpq*, *ppt2*, *ncf1*, *xcr1*, and *xcr1.1* at one or both timepoints. Vaccinated fish showed only a modest gene-level increase centered on day 3, with little effect on the overall module score. The MHCI antigen-processing module also increased on day 3 and day 7 in naïve fish (module scores +0.80 and +0.77), with coordinated rises in *psmb8*, *psmb9*, *tapbp.1*, *xbp1*, and several *mhci-u* paralogues. This module was notably heterogeneous among individual naïve fish. Vaccinated fish again showed only a small, transient elevation at day 3 at the level of individual genes, without a strong shift of the MHCI antigen-processing module. In conclusion, the DC-like cells in naïve fish showed increased antigen-processing activity concurrent with an increase in TLR response on day 3 and day 7.

Both activation/oxidative-response and antimicrobial-effector module scores in neutrophils showed a clear peak at day 3 in naïve and vaccinated fish (**Figure 6C**). Naïve fish reached module scores of +1.36 (activation/oxidative response) and +1.02 (antimicrobial effectors), with positive z-scores across multiple activation and effector genes including *adam8.1*, *cxcr2.1*, *arpc2*, *prdx2*, *bpi*, *hamp*, and *ctsl.4* (**Figure 6D**). Vaccinated fish exhibited a very similar pattern, with slightly higher day 3 module scores (+1.62 and +1.42) and broadly concordant gene-level increases (**Figures 6C, D**). In the serum-transfer experiment, both naïve-serum and immune-serum recipients showed a modest rise at day 1 (for example positive z-scores for *adam8.1*, *arpc2*, *bpi*, *hamp*, and *loc115550932/aerolysin-like protein*), and a small module induction.

qRT-PCR analysis showed expression levels of *bpi* in spleen and gills and *nccrp1* in spleen (**Figure 6E**), two genes that in the splenic scRNA-seq dataset are expressed almost exclusively by neutrophils. In spleen, naïve fish showed a strong day-3 induction of *bpi*, with two high-responders reaching ~1,700- and ~2,300-fold and the third fish, with low pathogen-burden, increasing only ~93-fold. By day 7, naïve spleen values were 140–800-fold. Vaccinated fish showed a similar rise at day 3 (80-580-fold) but a faster reduction at day 7 (4.8-28-fold). In gills, *bpi* expression at day 3 and day 7 was broadly similar in naïve and vaccinated fish. Naïve fish showed a split response in *bpi* expression in gills, with high responders (115- and 130-fold at day 3, 66-fold at day 7) and low responders (12-fold at day 3, 15-fold at day 7). Vaccinated fish showed a similar range of values across individuals at both day 3 and day 7. For *nccrp1* in spleen, naïve fish showed modest but consistent induction at day 3 (~2.2–2.7-fold) and day 7 (~4.0–7.6-fold). Vaccinated fish were markedly heterogeneous at day 3, with one fish rising to ~8.9-fold and the others remaining ~1.3–1.5-fold; by day 7 post infection, the levels of *nccrp1* expression in vaccinated cod were near baseline (~0.39–1.45-fold). In the splenic scRNA-seq heatmap, *nccrp1* expression remained low at day 3 in naïve fish, contrasting with the qRT-PCR increase. This likely reflects the different quantities captured by each assay: scRNA-seq measures mean per-cell expression within neutrophils, whereas qRT-PCR measures total transcript abundance in bulk tissue, integrating both expression per cell and changes in neutrophil abundance. In conclusion, we found strong but transient activation and antimicrobial activity in neutrophils on day 3 in both naïve and vaccinated fish.

## 4 Discussion

In this study, we used single-cell transcriptomics, supported by qRT-PCR and survival data, to characterize immune responses and pathogen control following bath infection of Atlantic cod with *V. anguillarum*. Immune signatures from actively bath-vaccinated cod and cod receiving passive immunization via transfer of immune serum were compared with appropriate control fish. By combining qRT-PCR-based quantification of bacterial burden with splenic single-cell profiling, we show that protection correlates with early restriction of systemic infection and reduced inflammatory activation. In addition, protected fish display a greater expansion of an activated B-cell population than naïve controls. These immune signatures, together with improved survival, were observed in both actively vaccinated fish and immune-serum recipients, indicating that antigen-specific IgM is a major protective factor, consistent with recent serum-transfer studies (7).

### 4.1 Mucosal containment and limited systemic replication in vaccinated fish

Pathogen burden quantified by qRT-PCR revealed a clear divergence between naïve and vaccinated fish from day 3 (**Figure 3**). Naïve fish exhibited high 16S rRNA gene levels and repeated *toxR* detection in spleen and head kidney, consistent with systemic *V. anguillarum* replication. Vaccinated fish maintained low systemic 16S rRNA gene and remained *toxR*-negative except for a single low spleen value. Occasional 16S rRNA gene level spikes in vaccinated head kidney without *toxR* detection likely represent phagocytosed bacterial material rather than viable infection.

Gills showed elevated 16S rRNA gene levels and sporadic *toxR* presence in both groups, reflecting surface exposure rather than internal spread. These molecular signatures align with survival outcomes: all vaccinated fish survived, whereas half of the naïve group died. Immune-serum recipients displayed the same containment phenotype, demonstrating that antigen-specific IgM alone is sufficient to block systemic dissemination (7). The divergent bacterial loads and immune responses among individual naïve fish are therefore best explained by variable success in early containment of infection, in contrast to the uniform mucosal restriction seen in vaccinated fish and immune-serum recipients.

### 4.2 Innate myeloid responses track pathogen burden

Naïve fish mounted strong and sustained innate myeloid activation that closely paralleled their high systemic *V. anguillarum* burden. Macrophages showed pronounced induction of *il1b*, *ctsl.4* and *ccl20* together with high inflammation-module scores (**Figure 2**), consistent with earlier work showing similar gene induction in cod tissues stimulated with *Vibrio* antigens (20–23). All groups exhibited an increase in splenic macrophage abundance following challenge (**Figure 5A**), in line with well-described recruitment and expansion of monocyte–macrophage populations during acute bacterial exposure in both fish and mammals (24–26). However, naïve fish showed a prolonged expansion of macrophages accompanied by sustained transcriptional activation, mirroring their elevated pathogen loads. The macrophages of vaccinated fish and immune-serum recipients returned toward baseline more rapidly and displayed minimal induction of inflammatory pathways.

DC-like cells displayed a similarly burden-linked immune signature (**Figures 6A, B**). Naïve fish showed coordinated reductions in *tlr13* paralogues, *tlr3*, *unc93b1*, and *cnpy3*, genes involved in endosomal nucleic-acid sensing and TLR trafficking, a pattern consistent with reports of PRR downregulation during acute bacterial or LPS stimulation in other teleosts (27, 28) and with known TLR negative-feedback regulation in mammals (29). Despite this early PRR suppression, naïve fish mounted strong downstream activation, including induction of antimicrobial and phagocyte-associated genes (*mpeg1*, *ncf1*, *cpq*), DC-associated chemokine receptor *xcr1* paralogues, and enhanced MHCI antigen-processing and presentation machinery (*psmb8*, *psmb9*, *tapbp.1*, *xbp1*, and multiple *mhc1-u* loci). These responses were heterogeneous and aligned with the split in systemic pathogen burden in different fish revealed by 16S rRNA gene and *toxR*. In contrast, vaccinated and immune-serum-recipient fish showed only weak or transient DC-like activation. Collectively, the coordinated induction of PRR-associated sensing pathways, downstream inflammatory effector programs, and MHCI antigen-processing machinery supports the assignment of this cluster as DC-like, first described by Guslund et al. (8), with transcriptional features consistent with antigen-presenting programs. In Atlantic cod, which lack CD4 and MHC class II, such DC-like cells are unlikely to fulfill canonical CD4^+^ T-cell priming functions and instead are likely to contribute to innate sensing, MHCI-restricted antigen presentation, and broader immune orchestration.

Neutrophils showed a different temporal profile but exhibited a similar burden-linked immune signature. All groups showed a rapid day-3 activation burst, with oxidative-response and antimicrobial-effector modules peaking to similar magnitudes (**Figures 6C, D**), consistent with the stereotyped early neutrophil response described in zebrafish infection models (30). Among antimicrobial genes, *bpi* and *nccrp1* were induced across groups (**Figure 6E**), in line with earlier reports of their upregulation following *V. anguillarum* antigen exposure in cod (20, 23). However, only naïve fish with high systemic pathogen burden exhibited more sustained *bpi* expression by qRT-PCR, while vaccinated fish returned toward baseline more quickly. The less consistent *nccrp1* patterns likely reflect modest per-cell expression combined with variation in neutrophil abundance captured by bulk qRT-PCR.

Together, these data indicate that antigen-specific IgM mounted by active vaccination or provided as a passive vaccine restricts systemic bacterial replication, thereby preventing the prolonged pathogen-associated molecular pattern (PAMP) exposure required to drive sustained activation of macrophages, DC-like cells, and neutrophils. Naïve fish, in contrast, experienced uncontained bacterial growth that resulted in prolonged myeloid activation across multiple innate lineages.

### 4.3 B cell activation is a consistent feature of response in both active and passive models

A striking feature of the dataset was the reproducible expansion of sub-cluster 1 B cells, a putative activated B-cell population (**Figures 4B, D**), across all groups, with a greater increase in vaccinated fish and immune-serum recipients compared with the two control groups (**Figure 5B**). This occurred without any rise in plasma-cell abundance and with stable transcript levels encoding secreted *igm* quantified by qRT-PCR. Transcriptionally, B-cell sub-cluster 1 showed elevated expression of *lbp*, which encodes a bactericidal-permeability-increasing/lipopolysaccharide-binding protein (BPI/LBP)-family protein related to mammalian LBPs, together with *tlr13* paralogues and the highest *ig* and *mhc1* transcript levels among non-plasma B-cell states. In other teleosts, BPI/LBP is implicated in binding lipopolysaccharide and modulating monocyte/macrophage responses to Gram-negative bacteria (31).

Given the absence of MHC class II and CD4 in cod (1, 2), these B cells are unlikely to reflect classical T cell–dependent B-cell responses. Instead, their transcriptional profile is consistent with activation pathways described for teleost B cells, including antigen uptake, phagocytic capacity, and direct pattern-recognition receptor signaling (32). Cod-specific studies similarly show that B cells possess strong phagocytic activity and robust innate signaling competence relative to mammalian counterparts (33), and cod maintain high baseline IgM titers throughout life (34). Together, these features support the idea that cod B cells can participate in early, T-independent responses to bacterial exposure.

The expansion of B-cell sub-cluster 1 across all groups likely reflects a general early-infection response. As Atlantic cod maintains high constitutive titers of broadly reactive IgM, it is plausible that even naïve fish generate low-affinity IgM–bacteria complexes during immersion exposure, although this has not been shown directly. By contrast, the amplified expansion in vaccinated fish and immune-serum recipients is most readily explained by the presence of pathogen-specific IgM, which has been shown to be protective against *V. anguillarum* in this system (7). A reasonable interpretation is that specific IgM enhances early bacterial binding and clearance, increasing the probability of B-cell encounters with IgM–bacteria complexes in the spleen and thereby augmenting activation through antigen uptake and PAMP-dependent signaling. Because a similar amplification occurs in vaccinated fish and immune-serum recipients, prior antigen experience of the responding B cells may not be required; instead, antigen-specific IgM may transiently reshape early B-cell activation dynamics. This remains hypothetical and will require targeted functional experiments. The increase in population size of B-cell sub-cluster 1 observed in immune-serum recipients was modest and overlapped with naïve-serum controls, indicating that any effect of passive antibody transfer in this model was limited. An additional or alternative explanation is that we missed the peak B-cell response, as the serum-transfer timeline lacked the day-3 sampling point where the strongest divergence was observed between the bath-vaccinated and naïve control fish. These interpretations should be viewed in the context of limited biological replication per timepoint (2–3 fish), which restricts the precision of population-level inference. Even so, the same overall pattern was observed across two independent experimental models, supporting the robustness of the qualitative conclusions.

The phenomenon of immune complexes being able to up- or downregulate antibody responses, depending on antibody isotype and other factors, is well known in mammals (35). A seminal study in mice showed that pre-existing natural IgM could shape the earliest stages of a primary B cell response, proposed to occur through the formation of antigen–IgM complexes that improved antigen handling and accelerated B-cell activation (36). Mice lacking secreted IgM showed delayed and less effective maturation of T-dependent IgG responses, and this defect was partly rescued by supplying natural IgM, indicating that baseline IgM can modulate early antigen availability and B-cell engagement. Although cod lack classical T-dependent pathways, the underlying principle – that pre-existing IgM giving rise to immune complexes may influence the initial quality and intensity of B-cell activation – provides a plausible parallel to the amplified sub-cluster 1 expansion observed in vaccinated fish and immune-serum recipients. In this context, our findings in cod reinforce the broader concept that IgM-mediated immune complex formation can play an important role in shaping early B-cell activation, a principle that may be more generally relevant across vertebrate immune systems than is often emphasized.

### 4.4 Absence of detectable T cell involvement

Across both infectious challenge experiments, neither the proportion of CD8^+^ T cells classified as activation ‘top responders’ nor the expression of T cell-associated transcripts showed meaningful evidence of T-cell activation (**Figure 5D**, **Supplementary Figure 7**). Expression of *cd3g* (pan-T-cell marker) and of *il18rap* and *ccl36* (markers of the CCL36^++^ T-cell subset) remained stable across timepoints in qRT-PCR data, and none of the transcriptionally defined splenic T-cell subsets showed detectable changes in abundance in scRNA-seq data. Cod also possess expanded MHCI gene families (2, 3, 37), supporting the view that cod adaptive immunity is organized differently from classical helper T cell-driven models. Given that the pathogenesis of *V. anguillarum* infection is primarily extracellular, it is mechanistically probable that protection is mediated primarily by IgM and phagocyte-driven pathways rather than CD8^+^ cytotoxic T-cell activity, which is typically associated with defense against intracellular pathogens. The ability of immune serum alone to confer protection is therefore consistent with the absence of a detectable T-cell response in this system.

### 4.5 Experimental scope and analytical considerations

Several aspects of the present study should be considered when interpreting the findings. Single-cell transcriptomic profiling was restricted to the spleen, which was selected as the primary secondary lymphoid organ in teleosts and because extensive reference scRNA-seq data are already available for Atlantic cod, enabling robust cell-type identification. Nonetheless, this focus excludes scRNA-seq analysis on other immunologically relevant compartments, including head kidney and mucosal tissues, where additional or distinct immune responses may occur. In the present study, we did not detect increased antibody production, plasma-cell expansion, or differential T-cell responses among experimental groups within the spleen; however, this does not preclude such activity occurring in other tissues.

Conclusions regarding the absence of detectable T-cell involvement are based on negative findings obtained using the analytical frameworks applied here, including assessment of population abundance, targeted qRT-PCR of selected T-cell–associated transcripts, and identification of highly activated cells using a cytotoxic/IFN-γ–associated gene module. While these approaches are suited to detecting canonical T-cell activation patterns in model systems, they may not capture alternative or non-classical modes of T-cell response, particularly in teleost-specific populations such as CCL36^+^ T cells, whose functional states and activation markers remain incompletely defined. Accordingly, the absence of a detectable splenic T-cell signal should be interpreted in the context of current limitations in both analytical resolution and functional annotation of teleost T-cell biology.

Biological replication was reduced by the exclusion of low-quality scRNA-seq samples following stringent quality control, and the infection timeline was not fully matched across experimental models. In particular, day 3 post-challenge samples in the bath-vaccination experiment were obtained from a repeat experiment performed the following year, and no corresponding day 3 time point was available for the serum-transfer study. These factors introduce potential sources of variability, including batch effects and inter-experimental differences. However, the central qualitative patterns observed across independent experimental models, together with concordant qRT-PCR measurements of pathogen burden and host responses, support the robustness of the main conclusions. Observations regarding the relative expansion or activation of specific immune cell populations, including B-cell subsets, should therefore be interpreted as qualitative patterns rather than statistically supported group-level differences, given the limited number of biological replicates and substantial inter-individual variability.

Functional interpretation of single-cell data relied on manually curated gene modules rather than automated pathway enrichment tools. Although this approach carries inherent limitations and requires informed biological judgment in gene selection, it reflects a deliberate choice necessitated by the absence of Atlantic cod from standard pathway databases and by extensive gene duplication and cod-specific immune gene expansions. Gene modules were constructed based on coherent expression patterns and established immune functions, with selection guided by established literature findings and comparative annotation rather than *post hoc* outcome-driven filtering. This module-based strategy prioritizes biological interpretability and internal consistency over automated enrichment frameworks that incompletely represent cod immune biology.

### 4.6 IgM-mediated containment shapes inflammatory outcomes

Together, these findings support a model in which protection in Atlantic cod is associated with early restriction of *V. anguillarum* dissemination, accompanied by reduced systemic inflammatory responses. Although splenic macrophage abundance increased following challenge in all groups, only naïve fish exhibited strong and sustained inflammatory activation, closely tracking their higher pathogen burden. In contrast, vaccinated fish and immune-serum recipients displayed limited and transient myeloid activation, despite comparable early neutrophil responses. Neutrophils mounted a rapid, stereotyped effector response across groups, but this response did not distinguish protected from unprotected states. Collectively, these observations indicate that the magnitude and duration of splenic myeloid activation primarily reflect pathogen burden rather than serving as the principal determinant of protective outcome.

Beyond these myeloid-associated patterns, our findings suggest that B cells contribute to protection in cod through two separable, IgM-linked roles. First, vaccination-induced, antigen-specific IgM promotes early mucosal containment of *V. anguillarum*, limiting systemic dissemination and downstream inflammatory activation. Second, the presence of antigen-specific IgM appears to amplify an early B-cell activation state that does not require cognate B-cell receptor engagement. This suggests that IgM can shape early cellular immune responses not only through direct antimicrobial activity, but also by modulating B-cell behavior during acute infection. Such antibody-enabled recruitment of innate-like B-cell functions aligns with known properties of teleost B cells and points to a broader principle in which antibodies act as organizers of early immune responses. In this context, vaccine efficacy may depend not only on antibody production, but also on how antibody responses influence early immune organization.

Future work should address the functional capacities of the activated B-cell subset, including phagocytosis, antigen uptake, and interactions with opsonized bacteria. Early gill responses also appear central to determining infection outcome, highlighting the need for mucosal transcriptomics or spatial profiling to understand how containment is achieved.

## Data Availability

The raw sequencing data generated in this study are available in the European Nucleotide Archive (ENA) under study accession number ERP132119. Previously published datasets re-analysed in this study are available under accession number PRJEB47815.

## Supplementary material

Supplementary Figure 1 Identification of ambient RNA, which is shown in bold. **(A)** Heatmap showing top 10 differentially expressed genes for each cell cluster. Each row represents a gene and each column represents a cell cluster. **(B)** Violin plot showing the number of genes per cell expressed by each cell population.

Supplementary Figure 2 Identification and removal of erythroid and thrombocyte contaminants from the B-cell dataset. **(A)** UMAP embedding of 18, 893 splenic B cells from the active and passive vaccination experiments. Each point represents a single cell colored by transcriptionally defined cluster identity. **(B)** Lineage module scores were calculated for curated gene sets representing: B-cell identity: *bcl11a, pax5, cd79a, cd79b, cd22.18, blnk*, and *bank1*. Erythroid lineage: *hba, hba.1, hbb.2, hbb.3, slc4a1.1, rhag, epb41.1, tal1*, and *prdx2*. Thrombocyte lineage: *gp1ba, itga2b, itgb3.1, mpl, thbs1, tln1, tpm1*, and *myh11*. Violin plots show module score distributions across clusters. Clusters 6 and 7 exhibit high erythroid or thrombocyte scores, indicating non-B-cell contamination. These 266 cells (1.6% of the total B-cell dataset) were removed prior to downstream analyses.

Supplementary Figure 3 Integration of day-3 samples (NaïvD3 and VacD3) into the combined scRNA-seq dataset. Uniform Manifold Approximation and Projection (UMAP) of the integrated spleen single-cell transcriptomes (CCA reduction), showing all cells from the active and passive vaccination experiments. Gray points represent all non-day-3 cells. Cells originating from the additional day-3 cohort (NaïvD3 in blue and VacD3 in orange) are overlaid at increased point size to highlight their distribution. NaïvD3 and VacD3 cells lie directly within the existing transcriptional structures across all major immune and non-immune lineages, demonstrating that the separately collected day-3 samples integrate consistently with the rest of the dataset after CCA-based batch correction.

Supplementary Figure 4 Survival of Atlantic cod following infection with *Vibrio anguillarum* O2a after either bath vaccination or passive serum transfer. This figure is adapted from **Figure 2** in Jonsson et al. (7). Continuous lines show survival of bath-vaccinated fish (Vac, n = 20, orange) and naïve fish (Naïv, n = 21, blue). Dotted lines show survival of serum-transfer recipients injected 24 hours before challenge with either naïve serum (NaïS, n = 26, black) or immune serum (ImmS, n = 27, purple). Survival differences were analyzed using the Mantel–Cox test; ****p < 0.0001.

Supplementary Figure 5 Slingshot pseudotime ordering of B-cell sub-clusters. **(A)** Cells from Seurat B-cell clusters 0–5 were projected in the selected low-dimensional embedding (umap.cca) and ordered along an inferred trajectory using Slingshot. Pseudotime values are min–max scaled to 0–1 and shown per cell (color scale). Solid curves indicate the fitted Slingshot lineage(s); sub-cluster 5 (plasma-cell cluster) was specified as the terminal state. The lineage orders are: Lineage 1: 2 -> 0 -> 1 -> 3 -> 4. Lineage 2: 2 -> 0 -> 1 -> 3 -> 5. **(B)** Distribution of scaled pseudotime values across clusters 0–5, shown as violin plots with overlaid boxplots (center line, median; box, interquartile range; whiskers, 1.5× IQR).

Supplementary Figure 6 Relative proportions of B cell sub-clusters 0, 2 and 3 in Atlantic cod spleen across active vaccination and serum-transfer challenge timelines. Panels show the same timeline layout: active vaccination on the left and serum-transfer on the right. Fish were sampled before challenge (baseline) and at days (D) 1, 3, 7, and 15 post-challenge in the active vaccination experiment, and at day 0 and days 1, 7, and 15 post-challenge in the serum-transfer experiment. Each symbol represents an individual fish, colored and shaped by experimental group (baseline: gray circle; naïve: blue triangle; vaccinated: yellow square; naïve-serum recipients: teal pentagon; immune-serum recipients: purple diamond). Horizontal bars indicate group medians. **(A)** Percentage of sub-cluster 0 among all splenic B cells. **(B)** Percentage of sub-cluster 2 among all splenic B cells. **(C)** Percentage of sub-cluster 3 among all splenic B cells.

Supplementary Figure 7 Relative proportions of T cell sub-clusters in Atlantic cod spleen across active vaccination and serum-transfer challenge timelines. Panels show the same timeline layout: active vaccination on the left and serum-transfer on the right. Fish were sampled before challenge (baseline) and at days (D) 1, 3, 7, and 15 post-challenge in the active vaccination experiment, and at day 0 and days 1, 7, and 15 post-challenge in the serum-transfer experiment. Each symbol represents an individual fish, colored and shaped by experimental group (baseline: gray circle; naïve: blue triangle; vaccinated: yellow square; naïve-serum recipients: teal pentagon; immune-serum recipients: purple diamond). Horizontal bars indicate group medians. **(A)** Percentage of CCL36^+^ T cells among all splenic T cells. **(B)** Percentage of γδ T cells among all splenic T cells. **(C)** Percentage of CD8+ T cells among all splenic T cells. **(D)** Percentage of proliferating T cells among all splenic T cells. **(E)** Percentage of CCL36^++^ T cells among all splenic T cells.

Supplementary Figure 8 T cell marker mRNA expression in spleen, head kidney (HK), and gills as measured by qPCR. Expression of *cd3g* (pan-T-cell marker) is shown in the top row, followed by the CCL36^++^ T-cell–associated genes *ccl36* and *il18rap* in the middle and bottom row respectively. Expression level is shown relative to host *eef1a1* (log_10_ scale) at days (D) 0, 1, 3 and 7 post-infection in naïve (blue triangles) and vaccinated (yellow squares) fish. Each point represents one fish, and bars indicate medians. Data are from the active vaccination experiment.

Supplementary Figure 9 B cell marker mRNA expression in spleen, head kidney (HK), and gills as measured by qPCR. Expression of *cd22* is shown in the top row, followed by *cd79a* in the middle row, and secreted IgM [(S)*igm*]) in the bottom row. Expression level is shown relative to host *eef1a1* (log_10_ scale) at days (D) 0, 1, 3 and 7 post-infection in naïve (blue triangles) and vaccinated (yellow squares) fish. Each point represents one fish, and bars indicate medians. Data are from the active vaccination experiment.

Supplementary Figure 10 Expression of macrophage-associated genes (*il1b, ccl20, ctsl.4*) in gills as measured by qPCR. Expression level is shown relative to host *eef1a1* (log_10_ scale) at days (D) 0, 1, 3 and 7 post-infection in naïve (blue triangles) and vaccinated (yellow squares) fish. Each point represents one fish, and bars indicate medians. Data are from the active vaccination experiment.

## References

[1] Pilström L Warr GW Strömberg S . Why is the antibody response of Atlantic cod so poor? The search for a genetic explanation. Fish Sci. (2005) 71:961–71. doi: 10.1111/j.1444-2906.2005.01052.x, PMID: 41769773

[2] Star B Nederbragt AJ Jentoft S Grimholt U Malmstrøm M Gregers TF . The genome sequence of Atlantic cod reveals a unique immune system. Nature. (2011) 477:207. doi: 10.1038/nature10342, PMID: 21832995 PMC3537168

[3] Malmstrøm M Jentoft S Gregers TF Jakobsen KS . Unraveling the evolution of the atlantic cod’s (*Gadus morhua* L.) alternative immune strategy. PloS One. (2013) 8(9):e74004. doi: 10.1371/journal.pone.0074004, PMID: 24019946 PMC3760826

[4] Espelid S Rødseth OM Jørgensen TØ . Vaccination experiments and studies of the humoral immune responses in cod, *Gadus morhua* L., to four strains of monoclonal-defined Vibrio Anguillarum. J Fish Dis. (1991) 14:185–97. doi: 10.1111/j.1365-2761.1991.tb00588.x, PMID: 41769773

[5] Magnadottir B Jonsdottir H Helgason S Bjornsson B Solem ST Pilstrom L . Immune parameters of immunised cod (*Gadus morhua* L.). Fish Shellfish Immun. (2001) 11:75–89. 10.1006/fsim.2000.0296 11271604

[6] Lund V Bordal S Schröder MB . Specificity and durability of antibody responses in Atlantic cod (*Gadus morhua* L.) immunised with Vibrio Anguillarum O2b. Fish Shellfish Immun. (2007) 23:906–10. doi: 10.1016/j.fsi.2007.04.006, PMID: 17604648

[7] Jonsson A López-Porras A Nørstebø SF Guslund NC Sørum H Qiao S-W . Protective IgM-mediated immunity against Vibrio Anguillarum in Atlantic cod with evolutionary losses of mhc class II and cd4. Front Immunol. (2025) 16. doi: 10.3389/fimmu.2025.1579541, PMID: 40672951 PMC12265950

[8] Guslund NC Solbakken MH Brieuc MSO Jentoft S Jakobsen KS Qiao SW . Single-cell transcriptome profiling of immune cell repertoire of the Atlantic cod which naturally lacks the major histocompatibility class II system. Front Immunol. (2020) 11:559555. doi: 10.3389/fimmu.2020.559555, PMID: 33154745 PMC7588623

[9] Guslund NC Krabberød AK Nørstebø SF Solbakken MH Jakobsen KS Johansen F-E . Lymphocyte subsets in Atlantic cod (*Gadus morhua*) interrogated by single-cell sequencing. Commun Biol. (2022) 5:689. doi: 10.1038/s42003-022-03645-w, PMID: 35821077 PMC9276791

[10] Frans I Michiels CW Bossier P Willems KA Lievens B Rediers H . *Vibrio anguillarum* as a fish pathogen: virulence factors, diagnosis and prevention. J Fish Dis. (2011) 34:643–61. doi: 10.1111/j.1365-2761.2011.01279.x, PMID: 21838709

[11] Macosko E Goldman M . Drop-seq laboratory protocol (2015). Available online at: http://mccarrolllab.org/dropseq/Online-Dropseq-Protocol-v.-3.1-Dec-2015.pdf (Accessed June, 2019).

[12] Macosko EZ Basu A Satija R Nemesh J Shekhar K Goldman M . Highly parallel genome-wide expression profiling of individual cells using nanoliter droplets. Cell. (2015) 161:1202–14. doi: 10.1016/j.cell.2015.05.002, PMID: 26000488 PMC4481139

[13] Namesh J . Drop­seq core computational protocol version 1.0.1 (2015). Available online at: http://mccarrolllab.org/wp-content/uploads/2016/03/Drop-seqAlignmentCookbookv1.2Jan2016.pdf (Accessed June 3, 2019).

[14] Dobin A Davis CA Schlesinger F Drenkow J Zaleski C Jha S . STAR: ultrafast universal RNA-seq aligner. Bioinformatics. (2013) 29:15–21. doi: 10.1093/bioinformatics/bts635, PMID: 23104886 PMC3530905

[15] Satija R Farrell JA Gennert D Schier AF Regev A . Spatial reconstruction of single-cell gene expression data. Nat Biotechnol. (2015) 33:495–502. doi: 10.1038/nbt.3192, PMID: 25867923 PMC4430369

[16] Butler A Hoffman P Smibert P Papalexi E Satija R . Integrating single-cell transcriptomic data across different conditions, technologies, and species. Nat Biotechnol. (2018) 36:411–20. doi: 10.1038/nbt.4096, PMID: 29608179 PMC6700744

[17] Stuart T Butler A Hoffman P Hafemeister C Papalexi E Mauck WM . Comprehensive integration of single-cell data. Cell. (2019) 177:1888–1902 e1821. doi: 10.1016/j.cell.2019.05.031, PMID: 31178118 PMC6687398

[18] Hao Y Hao S Andersen-Nissen E Mauck WM Zheng S Butler A . Integrated analysis of multimodal single-cell data. Cell. (2021) 184:3573–3587 e3529. doi: 10.1016/j.cell.2021.04.048, PMID: 34062119 PMC8238499

[19] Jentoft S Torresen OK Tooming-Klunderud A Skage M Kollias S Jakobsen KS . The genome sequence of the Atlantic cod, *Gadus morhua* (Linnaeus 1758). Wellcome Open Res. (2024) 9:189. doi: 10.12688/wellcomeopenres.21122.1, PMID: 39224768 PMC11367075

[20] Caipang CM Hynes N Puangkaew J Brinchmann MF Kiron V . Intraperitoneal vaccination of Atlantic cod, *Gadus morhua* with heat-killed Listonella Anguillarum enhances serum antibacterial activity and expression of immune response genes. Fish Shellfish Immunol. (2008) 24:314–22. doi: 10.1016/j.fsi.2007.11.018, PMID: 18226548

[21] Seppola M Larsen AN Steiro K Robertsen B Jensen I . Characterisation and expression analysis of the interleukin genes, IL-1beta, IL-8 and IL-10, in Atlantic cod (*Gadus morhua* L.). Mol Immunol. (2008) 45:887–97. doi: 10.1016/j.molimm.2007.08.003, PMID: 17875325

[22] Caipang CMA Brinchmann MF Kiron V . Profiling gene expression in the spleen of Atlantic cod, *Gadus morhua* upon vaccination with Vibrio Anguillarum antigen. Comp Biochem Phys A. (2009) 153:261–7. doi: 10.1016/j.cbpb.2009.03.005, PMID: 19318130

[23] Caipang CM Lazado CC Brinchmann MF Kiron V . Infection-induced changes in expression of antibacterial and cytokine genes in the gill epithelial cells of Atlantic cod, *Gadus morhua* during incubation with bacterial pathogens. Comp Biochem Physiol B Biochem Mol Biol. (2010) 156:319–25. doi: 10.1016/j.cbpb.2010.04.009, PMID: 20430108

[24] Pietras EM Miller LS Johnson CT O’Connell RM Dempsey PW Cheng G . A MyD88-dependent IFNγR-CCR2 signaling circuit is required for mobilization of monocytes and host defense against systemic bacterial challenge. Cell Res. (2011) 21:1068–79. doi: 10.1038/cr.2011.59, PMID: 21467996 PMC3193491

[25] Saleh M Montero R Kumar G Sudhagar A Friedl A Köllner B . Kinetics of local and systemic immune cell responses in whirling disease infection and resistance in rainbow trout. Parasites Vectors. (2019) 12:249. doi: 10.1186/s13071-019-3505-9, PMID: 31113489 PMC6528198

[26] Sarais F Montero R Ostermann S Rebl A Köllner B Goldammer T . The Early Immune Response of Lymphoid and Myeloid Head-Kidney Cells of Rainbow Trout (*Oncorhynchus mykiss*) Stimulated with Aeromonas salmonicida. Fishes. (2022) 7:12. doi: 10.3390/fishes7010012, PMID: 41725453

[27] Fan H Wang L Wen H Wang K Qi X Li J . Genome-wide identification and characterization of toll-like receptor genes in spotted sea bass (*Lateolabrax maculatus*) and their involvement in the host immune response to *Vibrio harveyi* infection. Fish Shellfish Immunol. (2019) 92:782–91. doi: 10.1016/j.fsi.2019.07.010, PMID: 31288100

[28] Sousa CSV Power DM Guerreiro PM Louro B Chen L Canário AVM . Transcriptomic Down-Regulation of Immune System Components in Barrier and Hematopoietic Tissues after Lipopolysaccharide Injection in Antarctic *Notothenia coriiceps*. Fishes. (2022) 7:171. doi: 10.3390/fishes7040171, PMID: 41725453

[29] Wang J Hu Y Deng WW Sun B . Negative regulation of Toll-like receptor signaling pathway. Microbes Infect. (2009) 11:321–7. doi: 10.1016/j.micinf.2008.12.011, PMID: 19146978

[30] Harvie EA Huttenlocher A . Neutrophils in host defense: new insights from zebrafish. J Leukoc Biol. (2015) 98:523–37. doi: 10.1189/jlb.4MR1114-524R, PMID: 25717145 PMC4569048

[31] Lu XJ Chu CQ Chen Q Chen J . A novel lipopolysaccharide-binding protein (LBP) gene from sweetfish *Plecoglossus altivelis*: molecular characterization and its role in the immune response of monocytes/macrophages. Fish Shellfish Immunol. (2014) 38:111–8. doi: 10.1016/j.fsi.2014.02.021, PMID: 24594008

[32] Li J Barreda DR Zhang YA Boshra H Gelman AE Lapatra S . B lymphocytes from early vertebrates have potent phagocytic and microbicidal abilities. Nat Immunol. (2006) 7:1116–24. doi: 10.1038/ni1389, PMID: 16980980

[33] Øverland HS Pettersen EF Rønneseth A Wergeland HI . Phagocytosis by B-cells and neutrophils in Atlantic salmon (Salmo salar L.) and Atlantic cod (*Gadus morhua* L.). Fish Shellfish Immun. (2010) 28:193–204. doi: 10.1016/j.fsi.2009.10.021, PMID: 19874896

[34] Magnadottir B Gudmundsdottir S Gudmundsdottir BK Helgason S . Natural antibodies of cod (*Gadus morhua* L.): Specificity, activity and affinity. Comp Biochem Phys B. (2009) 154:309–16. doi: 10.1016/j.cbpb.2009.07.005, PMID: 19631760

[35] Heyman B . Antibody feedback regulation. Immunol Rev. (2024) 328:126–42. doi: 10.1111/imr.13377, PMID: 39180190 PMC11659925

[36] Ehrenstein MR O’Keefe TL Davies SL Neuberger MS . Targeted gene disruption reveals a role for natural secretory IgM in the maturation of the primary immune response. Proc Natl Acad Sci United States America. (1998) 95:10089–93. doi: 10.1073/pnas.95.17.10089, PMID: 9707605 PMC21466

[37] Malmstrøm M Matschiner M Torresen OK Star B Snipen LG Hansen TF . Evolution of the immune system influences speciation rates in teleost fishes. Nat Genet. (2016) 48:1204–10. doi: 10.1038/ng.3645, PMID: 27548311

