# Supplementary material for: IgM-mediated protection drives early B-cell activation and mucosal containment of Vibrio anguillarum in Atlantic cod (Gadus morhua)
Source: Front Immunol. 2026 Mar 6;17:1771403. doi: 10.3389/fimmu.2026.1771403 (PMC13002412; doi:10.3389/fimmu.2026.1771403)
Supplement: Supplementary file 2 [file Table2.docx]

**Supplementary Table 2.** Primer sequences and performance metrics for quantitative PCR assays.

| **Target gene** | **Gene description** | **NCBI Gene ID / transcript** | **Forward primer (5′–3′)** | **Reverse primer (5′–3′)** | **Amplicon (bp)** | **Efficiency (%)** | **Reference / source** |
| --- | --- | --- | --- | --- | --- | --- | --- |
| *eef1a1* | Elongation factor 1-alpha 1 (reference) | LOC115536143 / XM_030347827.1 | TCCTTCAACGCCCAGGTCATC | CACGCTCTTGGGCAGATCCT | 168 | 98.9 | This study |
| *ccl36* | C-C motif chemokine 36 | LOC115529242 / XM_030337821.1 | GAACCTGTAACCATGCGTGC | CGGGACCACTTCCATTGTTG | 97 | 93 | This study |
| *bpi* | Bactericidal/permeability-increasing protein | LOC115556843 / XM_030374232.1 | GTCACCTTAAACCGGTTGGACA | ACACCACCCACTTGAGGACT | 106 | 100.9 | This study |
| *nccrp1* | Non-specific cytotoxic cell receptor protein 1 | LOC115560350 / XM_030360357.1 | GTGGACCTGAAGGCAGAAGG | TGGCTCTCCTCATACCAGTCT | 89 | 92.9 | This study |
| *il18rap* | Interleukin-18 receptor accessory protein | LOC115558504 / XM_030376693.1 | TGTGGCAATGAGAGTGGCG | GAAGGAGGGCAGTCAATCCC | 137 | 100.6 | This study |
| *il1b* | Interleukin-1β | LOC115546067 / XM_030359712.1 | GACCATGAGGCAAGTCGTCA | TCCAGCACCAGTTGTTCATCC | 147 | 89.8 | This study |
| *cd3g* | T-cell receptor CD3 gamma chain | LOC115560938 / XM_030380620.1 | ACCGCTGACAGTCAAAGCTC | TAAATGCCGTTGTCGACCCT | 77 | 96 | This study |
| *csf1rb* | Colony-stimulating factor-1 receptor b | LOC115547976 / XM_030362512.1 | CCACACACACTCCAAGGTGA | GCAGCAACGTCTCTGTGGAT | 128 | 94.6 | This study |
| *cd79a* | B-cell antigen receptor component alpha chain | LOC115550767 / XM_030370356.1 | CTGCAGGTCTACAAGCCGAT | AGCAGAAGCAGAATCCCCTG | 92 | 94.9 | This study |
| *cd22* | B-cell inhibitory receptor CD22 | LOC115554304 / XM_030370972.1 | TGCTGCTTAAAAGAGATTCCTTG | GAACACACTCGCCCTCTTTG | 87 | 99.4 | This study |
| *ccl20* | C-C motif chemokine 20 | LOC115537254 / XM_030349034.1 | TGTGTCAATGCCATCATTTTCCA | AGAGGAGCTACAGGGACGAG | 148 | 88.3 | This study |
| *ctsl* | Cathepsin L | LOC115544206 / XM_030357061.1 | GACTGGAGGGACAAGGGCTA | CCTCCAGAGAGCCTGTTGTG | 91 | 93.6 | This study |
| *Secreted igm* | Secreted immunoglobulin M | X58870 / Atlantic cod IgM mRNA | GAGCATCCACTGGCTCTTTA | GCAGCAAGCTATATCCAGGT | 97 | 94 | (Hori, Gamperl et al. 2010) |
| 16S rDNA | Bacterial 16S ribosomal RNA (*V. anguillarum*) | – (multiple operons; e.g. GenBank NC_015558.1) | CCACGCCGTAACGATGTCTA | CCAGGCGGTCTACTTAACGCGT | 81 | 107.4 | (Crisafi, Denaro et al. 2010) |
| toxR | Transcriptional regulator (*V. anguillarum*) | Locus tag VANGNB10_RS10950 (*V. anguillarum* NB10, NC_015558.1) | ACACCACCAACGAGCCTGA | TTGTCTCTTCGGGTTGCGA | 93 | 91.5 | (Crisafi, Denaro et al. 2010) |
