# Supplementary figures and images for: IgM-mediated protection drives early B-cell activation and mucosal containment of Vibrio anguillarum in Atlantic cod (Gadus morhua)
Source: Front Immunol. 2026 Mar 6;17:1771403. doi: 10.3389/fimmu.2026.1771403 (PMC13002412; doi:10.3389/fimmu.2026.1771403)

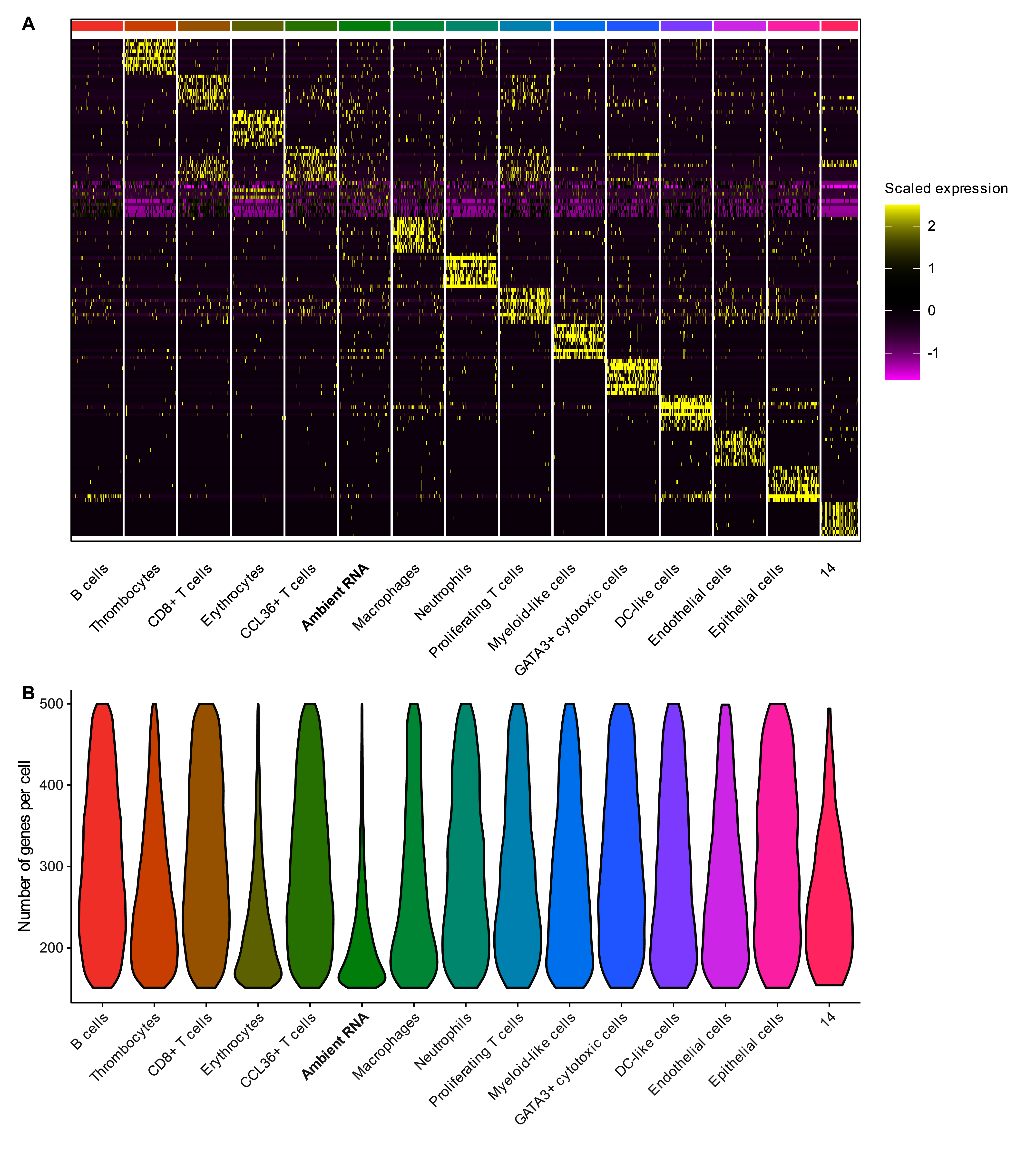

Supplement: Supplementary Figure 1 — Identification of ambient RNA, which is shown in bold. (A) Heatmap showing top 10 differentially expressed genes for each cell cluster. Each row represents a gene and each column represents a cell cluster. (B) Violin plot showing the number of genes per cell expressed by each cell population. [file Image1.tif]

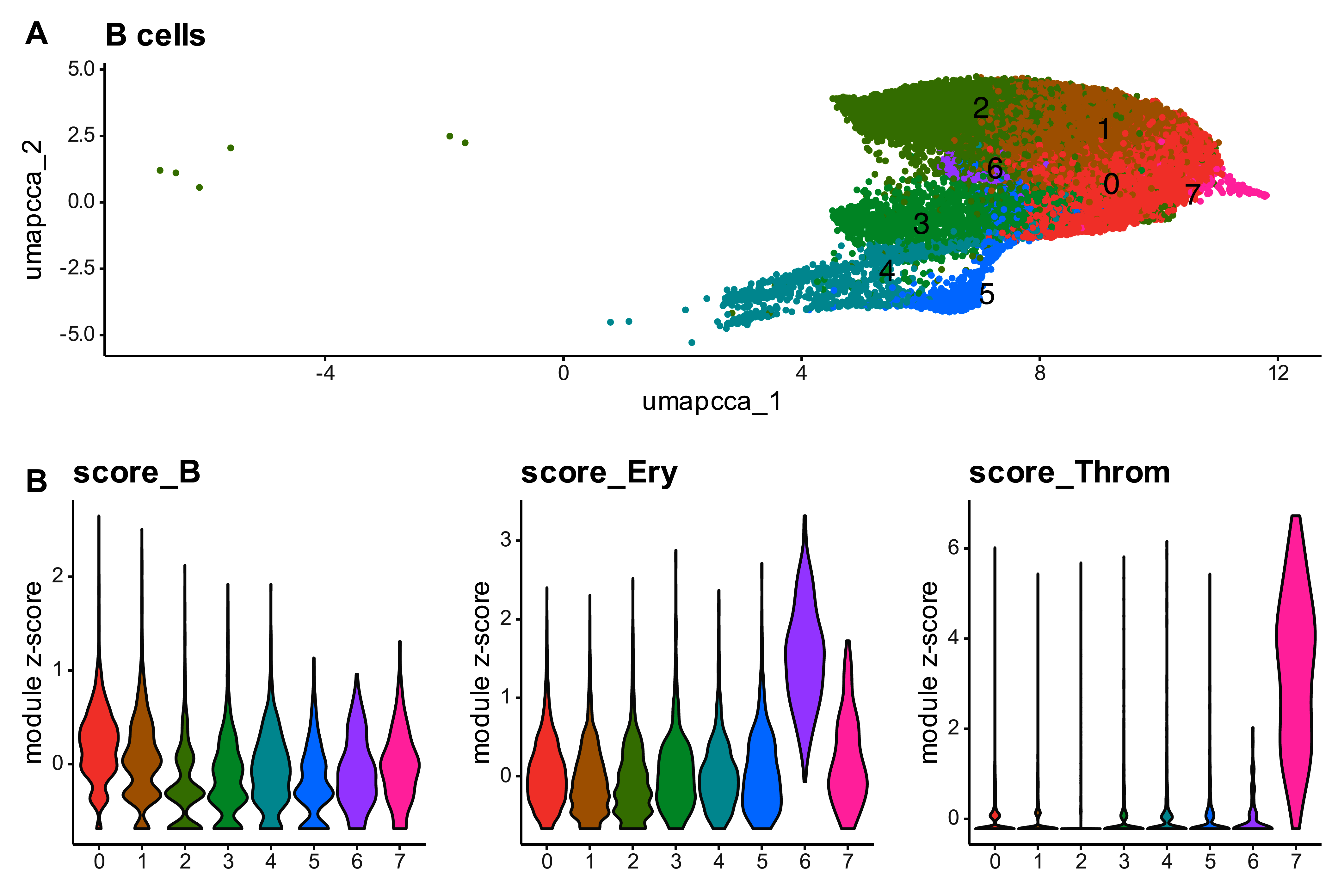

Supplement: Supplementary Figure 2 — Identification and removal of erythroid and thrombocyte contaminants from the B-cell dataset. (A) UMAP embedding of 18, 893 splenic B cells from the active and passive vaccination experiments. Each point represents a single cell colored by transcriptionally defined cluster identity. (B) Lineage module scores were calculated for curated gene sets representing: B-cell identity: bcl11a, pax5, cd79a, cd79b, cd22.18, blnk, and bank1. Erythroid lineage: hba, hba.1, hbb.2, hbb.3, slc4a1.1, rhag, epb41.1, tal1, and prdx2. Thrombocyte lineage: gp1ba, itga2b, itgb3.1, mpl, thbs1, tln1, tpm1, and myh11. Violin plots show module score distributions across clusters. Clusters 6 and 7 exhibit high erythroid or thrombocyte scores, indicating non-B-cell contamination. These 266 cells (1.6% of the total B-cell dataset) were removed prior to downstream analyses. [file Image2.tif]

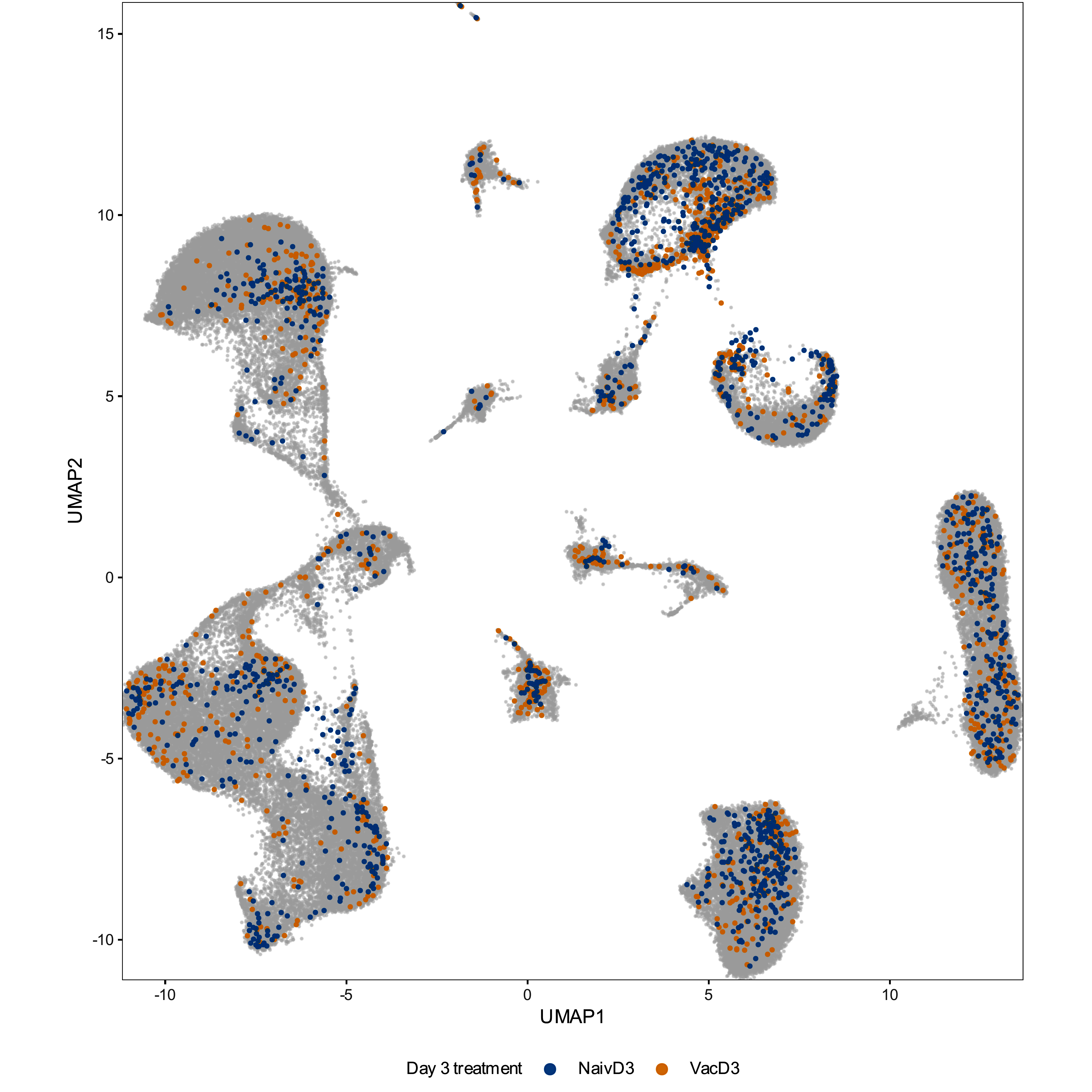

Supplement: Supplementary Figure 3 — Integration of day-3 samples (NaïvD3 and VacD3) into the combined scRNA-seq dataset. Uniform Manifold Approximation and Projection (UMAP) of the integrated spleen single-cell transcriptomes (CCA reduction), showing all cells from the active and passive vaccination experiments. Gray points represent all non-day-3 cells. Cells originating from the additional day-3 cohort (NaïvD3 in blue and VacD3 in orange) are overlaid at increased point size to highlight their distribution. NaïvD3 and VacD3 cells lie directly within the existing transcriptional structures across all major immune and non-immune lineages, demonstrating that the separately collected day-3 samples integrate consistently with the rest of the dataset after CCA-based batch correction. [file Image3.tif]

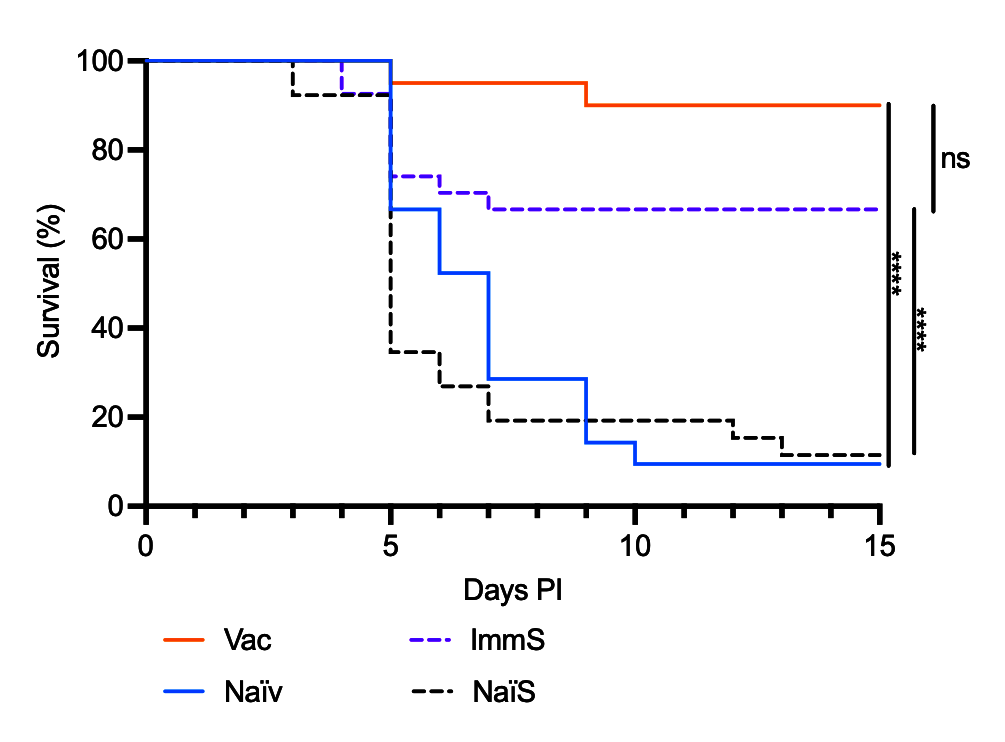

Supplement: Supplementary Figure 4 — Survival of Atlantic cod following infection with Vibrio anguillarum O2a after either bath vaccination or passive serum transfer. This figure is adapted from Figure 2 in Jonsson et al. (7). Continuous lines show survival of bath-vaccinated fish (Vac, n = 20, orange) and naïve fish (Naïv, n = 21, blue). Dotted lines show survival of serum-transfer recipients injected 24 hours before challenge with either naïve serum (NaïS, n = 26, black) or immune serum (ImmS, n = 27, purple). Survival differences were analyzed using the Mantel–Cox test; ****p < 0.0001. [file Image4.tif]

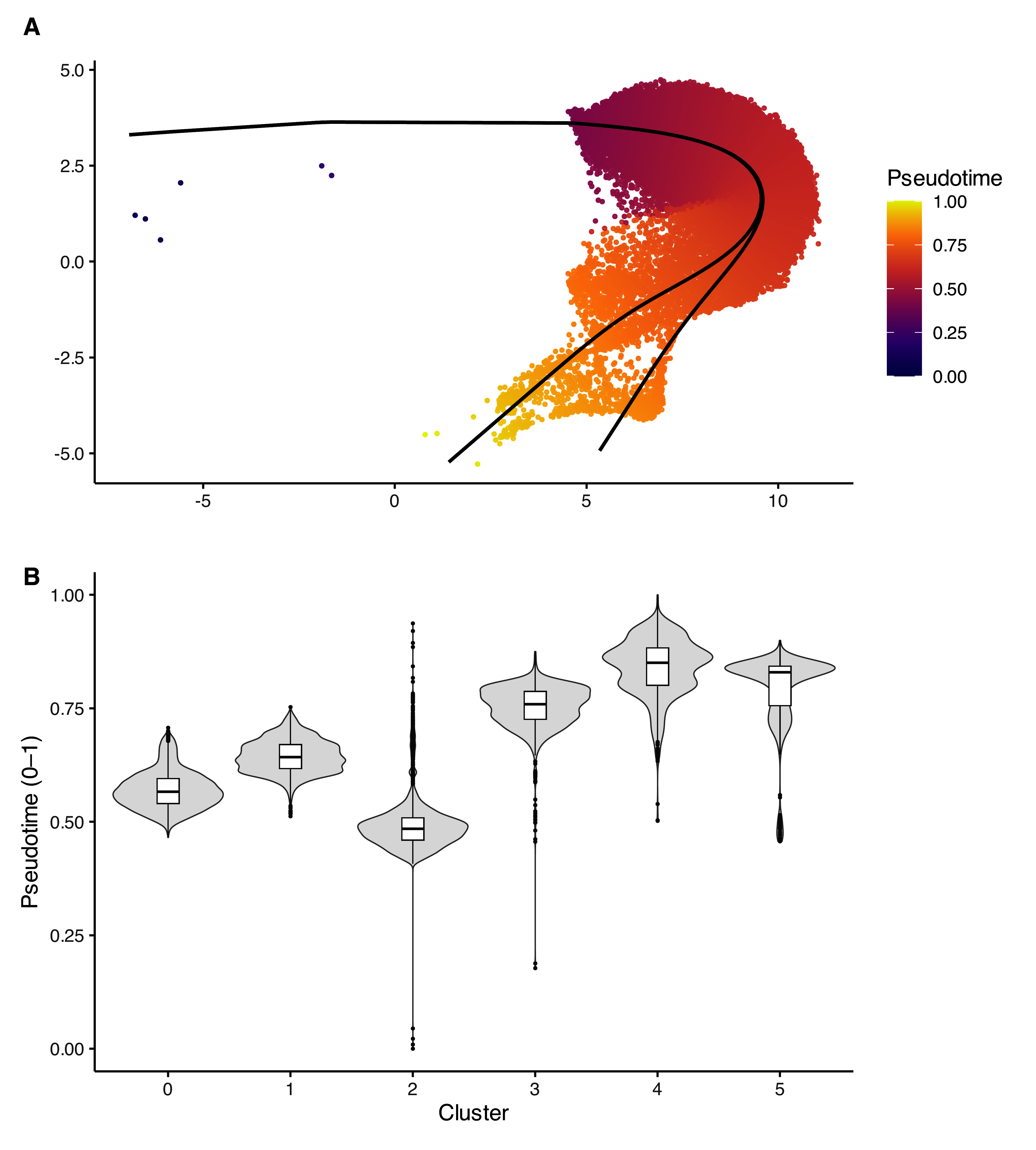

Supplement: Supplementary Figure 5 — Slingshot pseudotime ordering of B-cell sub-clusters. (A) Cells from Seurat B-cell clusters 0–5 were projected in the selected low-dimensional embedding (umap.cca) and ordered along an inferred trajectory using Slingshot. Pseudotime values are min–max scaled to 0–1 and shown per cell (color scale). Solid curves indicate the fitted Slingshot lineage(s); sub-cluster 5 (plasma-cell cluster) was specified as the terminal state. The lineage orders are: Lineage 1: 2 -> 0 -> 1 -> 3 -> 4. Lineage 2: 2 -> 0 -> 1 -> 3 -> 5. (B) Distribution of scaled pseudotime values across clusters 0–5, shown as violin plots with overlaid boxplots (center line, median; box, interquartile range; whiskers, 1.5× IQR). [file Image5.tif]

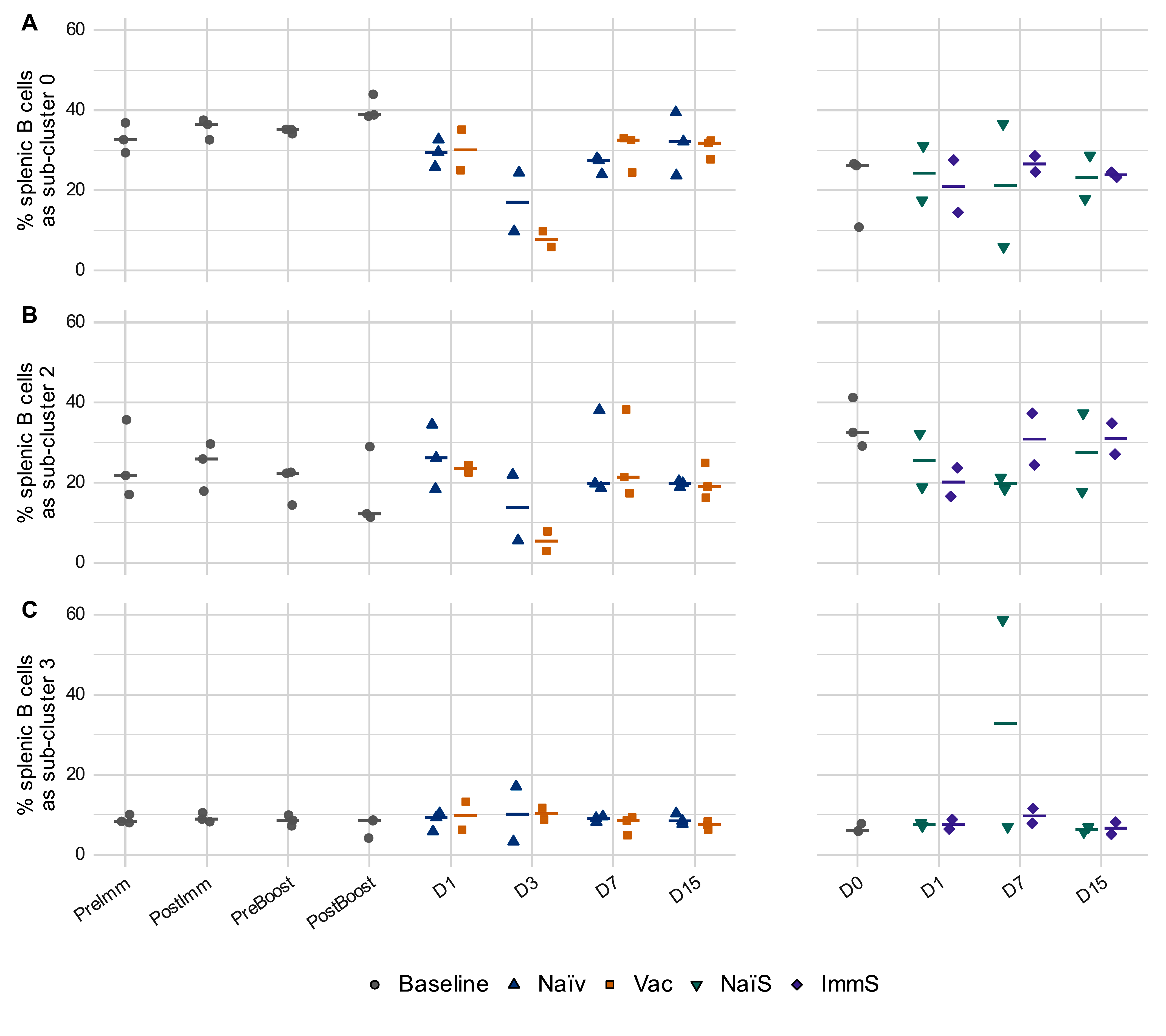

Supplement: Supplementary Figure 6 — Relative proportions of B cell sub-clusters 0, 2 and 3 in Atlantic cod spleen across active vaccination and serum-transfer challenge timelines. Panels show the same timeline layout: active vaccination on the left and serum-transfer on the right. Fish were sampled before challenge (baseline) and at days (D) 1, 3, 7, and 15 post-challenge in the active vaccination experiment, and at day 0 and days 1, 7, and 15 post-challenge in the serum-transfer experiment. Each symbol represents an individual fish, colored and shaped by experimental group (baseline: gray circle; naïve: blue triangle; vaccinated: yellow square; naïve-serum recipients: teal pentagon; immune-serum recipients: purple diamond). Horizontal bars indicate group medians. (A) Percentage of sub-cluster 0 among all splenic B cells. (B) Percentage of sub-cluster 2 among all splenic B cells. (C) Percentage of sub-cluster 3 among all splenic B cells. [file Image6.tif]

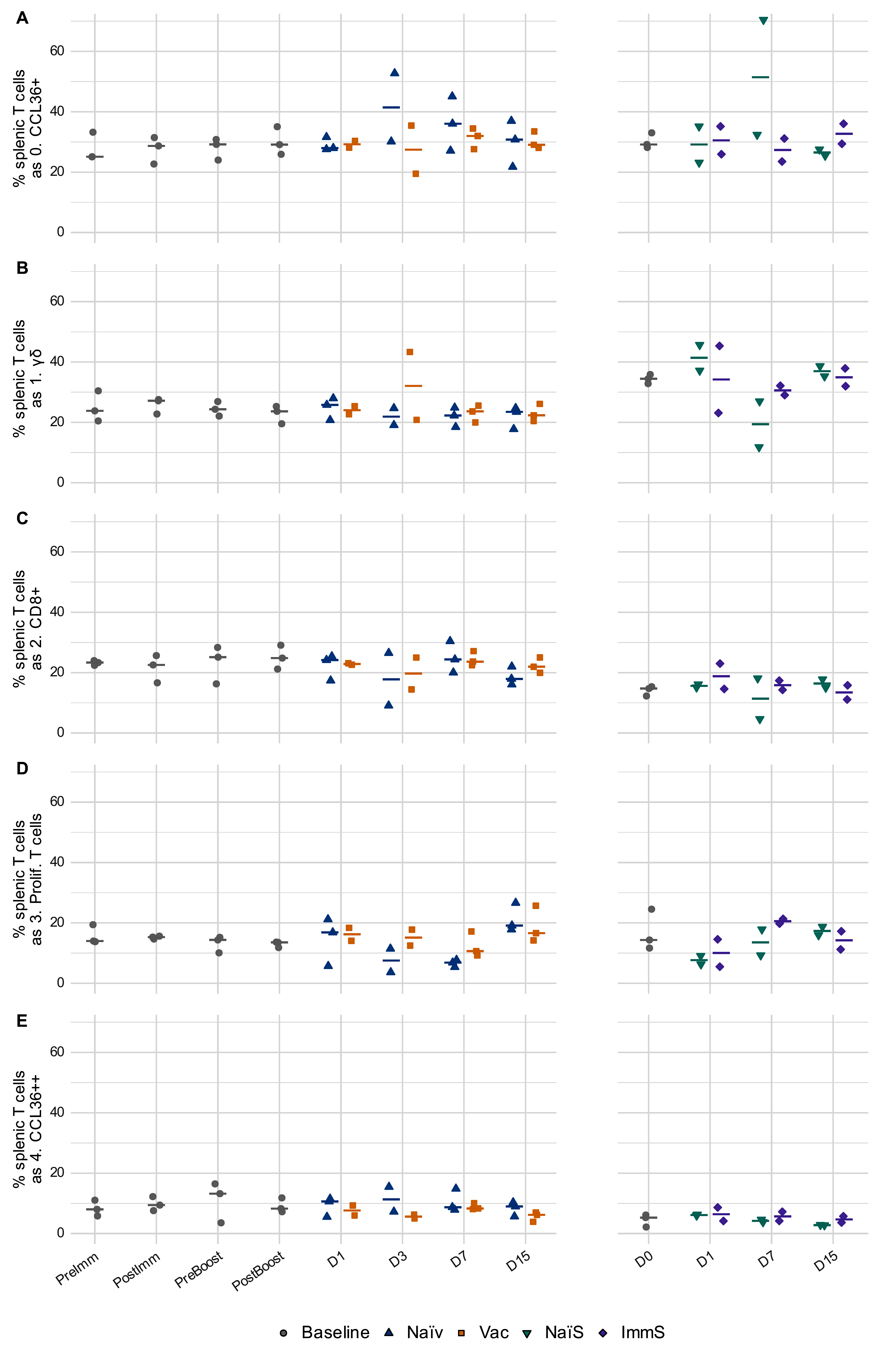

Supplement: Supplementary Figure 7 — Relative proportions of T cell sub-clusters in Atlantic cod spleen across active vaccination and serum-transfer challenge timelines. Panels show the same timeline layout: active vaccination on the left and serum-transfer on the right. Fish were sampled before challenge (baseline) and at days (D) 1, 3, 7, and 15 post-challenge in the active vaccination experiment, and at day 0 and days 1, 7, and 15 post-challenge in the serum-transfer experiment. Each symbol represents an individual fish, colored and shaped by experimental group (baseline: gray circle; naïve: blue triangle; vaccinated: yellow square; naïve-serum recipients: teal pentagon; immune-serum recipients: purple diamond). Horizontal bars indicate group medians. (A) Percentage of CCL36+ T cells among all splenic T cells. (B) Percentage of γδ T cells among all splenic T cells. (C) Percentage of CD8+ T cells among all splenic T cells. (D) Percentage of proliferating T cells among all splenic T cells. (E) Percentage of CCL36++ T cells among all splenic T cells. [file Image7.tif]

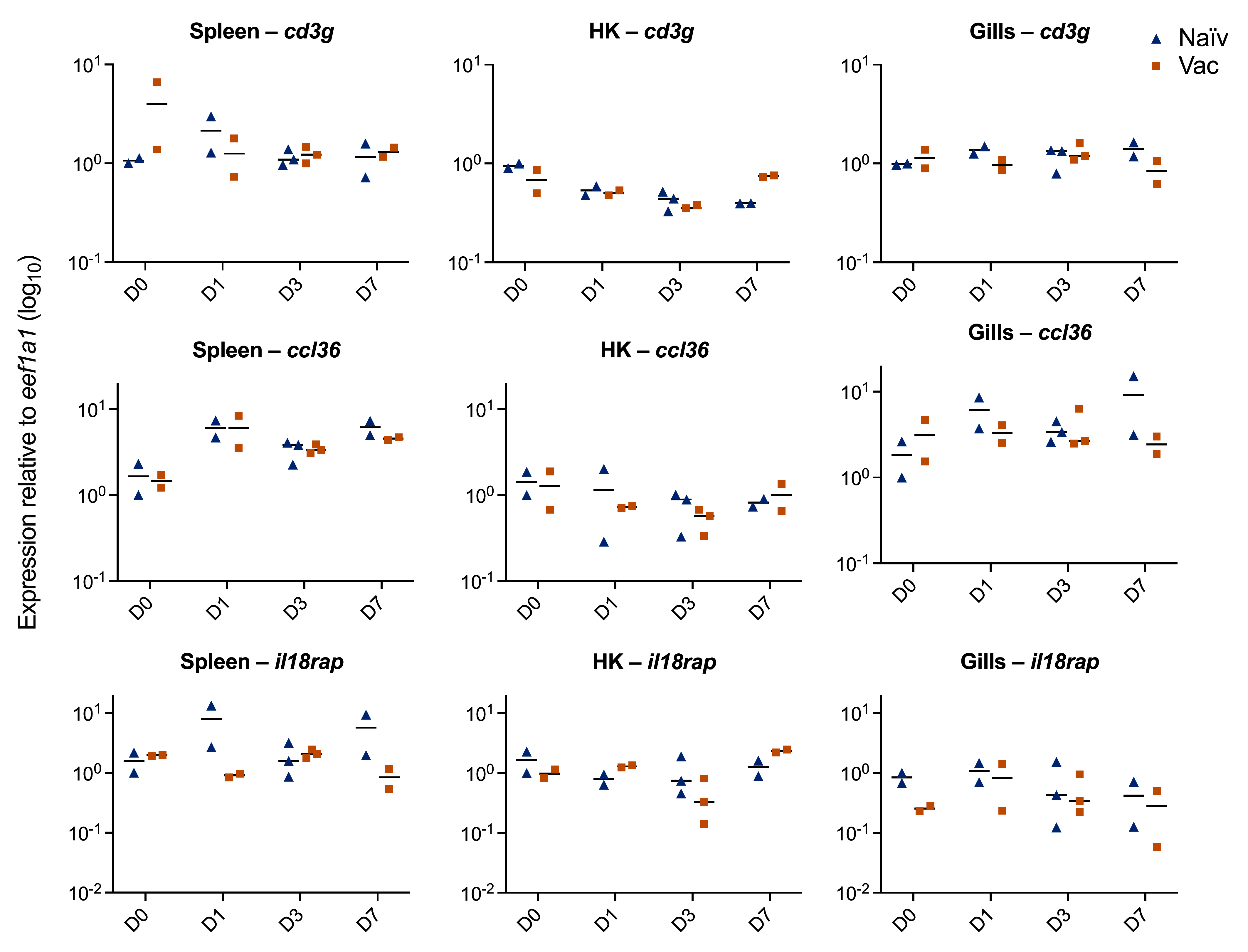

Supplement: Supplementary Figure 8 — T cell marker mRNA expression in spleen, head kidney (HK), and gills as measured by qPCR. Expression of cd3g (pan-T-cell marker) is shown in the top row, followed by the CCL36++ T-cell–associated genes ccl36 and il18rap in the middle and bottom row respectively. Expression level is shown relative to host eef1a1 (log10 scale) at days (D) 0, 1, 3 and 7 post-infection in naïve (blue triangles) and vaccinated (yellow squares) fish. Each point represents one fish, and bars indicate medians. Data are from the active vaccination experiment. [file Image8.tif]

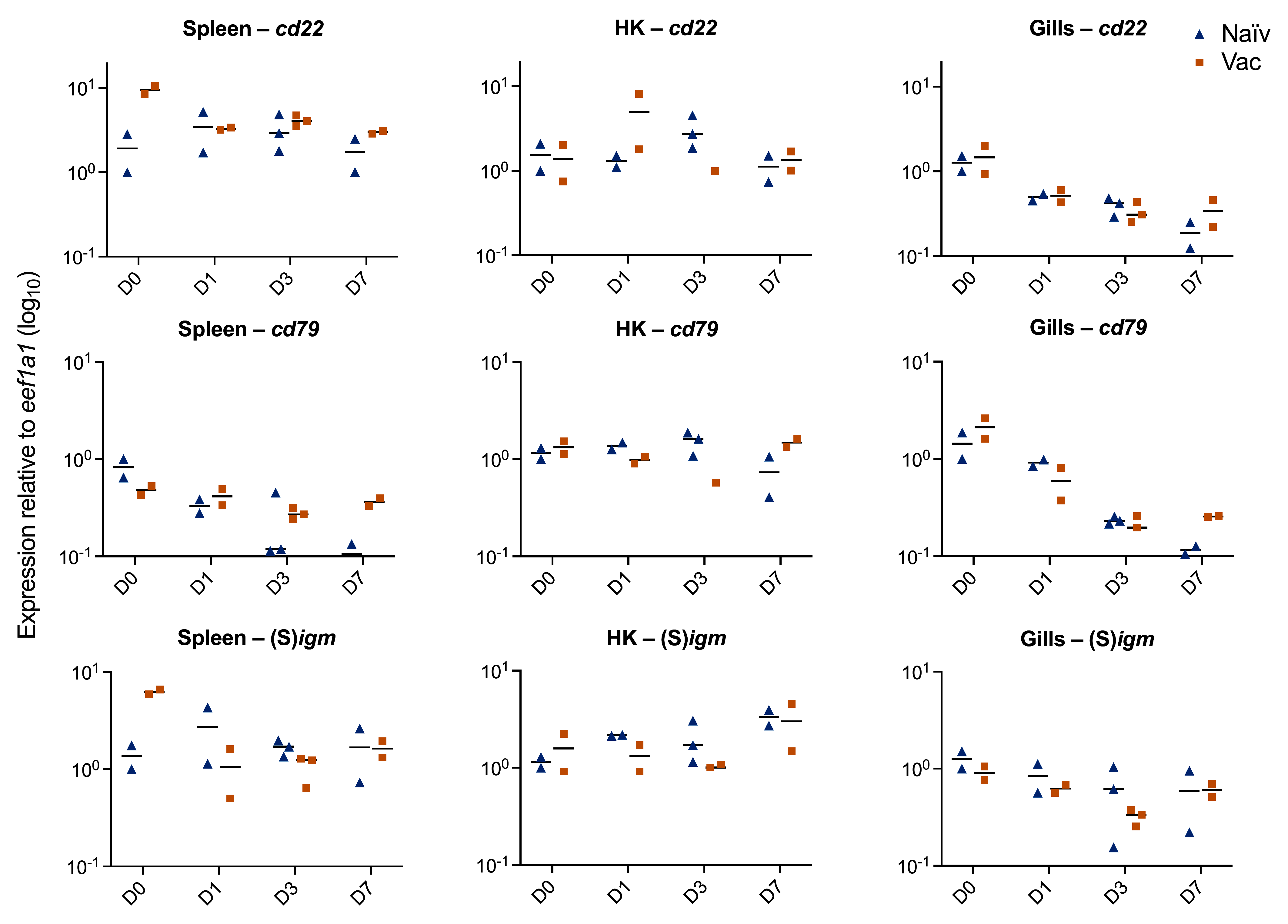

Supplement: Supplementary Figure 9 — B cell marker mRNA expression in spleen, head kidney (HK), and gills as measured by qPCR. Expression of cd22 is shown in the top row, followed by cd79a in the middle row, and secreted IgM [(S)igm]) in the bottom row. Expression level is shown relative to host eef1a1 (log10 scale) at days (D) 0, 1, 3 and 7 post-infection in naïve (blue triangles) and vaccinated (yellow squares) fish. Each point represents one fish, and bars indicate medians. Data are from the active vaccination experiment. [file Image9.tif]

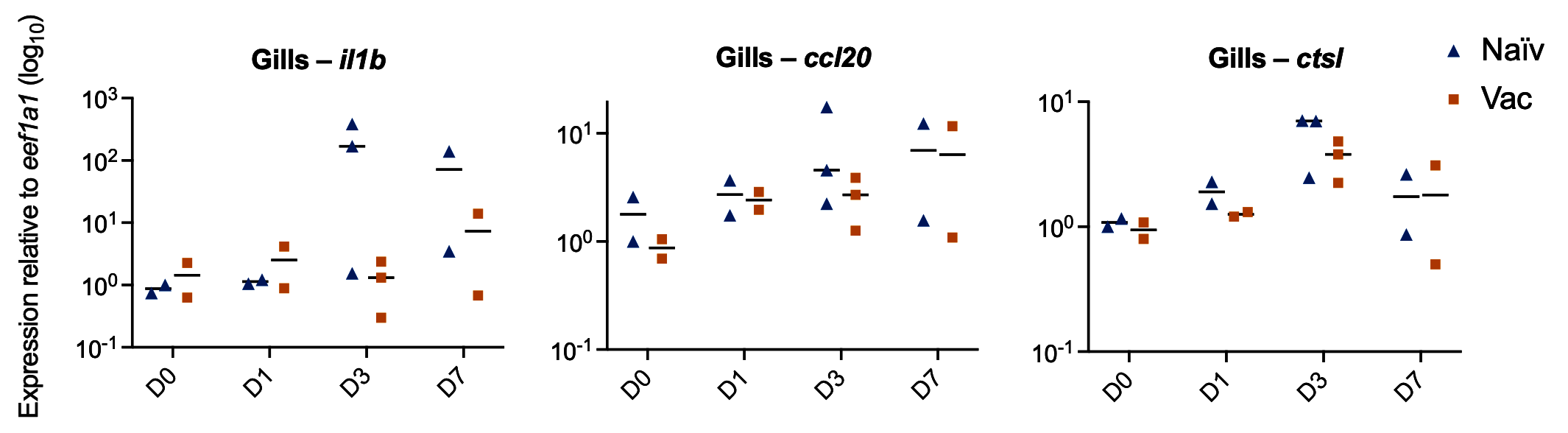

Supplement: Supplementary Figure 10 — Expression of macrophage-associated genes (il1b, ccl20, ctsl.4) in gills as measured by qPCR. Expression level is shown relative to host eef1a1 (log10 scale) at days (D) 0, 1, 3 and 7 post-infection in naïve (blue triangles) and vaccinated (yellow squares) fish. Each point represents one fish, and bars indicate medians. Data are from the active vaccination experiment. [file Image10.tif]
